# The 5S rDNA family evolves through concerted and birth-and-death evolution in fish genomes: an example from freshwater stingrays

**DOI:** 10.1186/1471-2148-11-151

**Published:** 2011-05-31

**Authors:** Danillo Pinhal, Tatiana S Yoshimura, Carlos S Araki, Cesar Martins

**Affiliations:** 1 UNESP - Universidade Estadual Paulista, Instituto de Biociências, Departamento de Morfologia, Botucatu, SP, Brazil

## Abstract

**Background:**

Ribosomal 5S genes are well known for the critical role they play in ribosome folding and functionality. These genes are thought to evolve in a concerted fashion, with high rates of homogenization of gene copies. However, the majority of previous analyses regarding the evolutionary process of rDNA repeats were conducted in invertebrates and plants. Studies have also been conducted on vertebrates, but these analyses were usually restricted to the 18S, 5.8S and 28S rRNA genes. The recent identification of divergent 5S rRNA gene paralogs in the genomes of elasmobranches and teleost fishes indicate that the eukaryotic 5S rRNA gene family has a more complex genomic organization than previously thought. The availability of new sequence data from lower vertebrates such as teleosts and elasmobranches enables an enhanced evolutionary characterization of 5S rDNA among vertebrates.

**Results:**

We identified two variant classes of 5S rDNA sequences in the genomes of Potamotrygonidae stingrays, similar to the genomes of other vertebrates. One class of 5S rRNA genes was shared only by elasmobranches. A broad comparative survey among 100 vertebrate species suggests that the 5S rRNA gene variants in fishes originated from rounds of genome duplication. These variants were then maintained or eliminated by birth-and-death mechanisms, under intense purifying selection. Clustered multiple copies of 5S rDNA variants could have arisen due to unequal crossing over mechanisms. Simultaneously, the distinct genome clusters were independently homogenized, resulting in the maintenance of clusters of highly similar repeats through concerted evolution.

**Conclusions:**

We believe that 5S rDNA molecular evolution in fish genomes is driven by a mixed mechanism that integrates birth-and-death and concerted evolution.

## Background

The nuclear ribosomal DNA (rDNA) is organized into two distinct multigene families comprising the so-called 45S and 5S rDNA repeats. The 45S rDNA repeats contain the genes that are transcribed into 18S, 5.8S and 26S-28S rRNA and spacers (IGS, ITS1 and ITS2), whereas 5S rDNA encodes the 5S rRNA transcribing region (120 bp long and highly conserved) and a variable nontranscribed spacer (NTS) [for a review, see 1]. A common characteristic of 5S rDNA is multiple tandemly arrayed repeats, at one or several chromosomal locations throughout the genome. Furthermore, the 5S rDNA has been reported to be linked to other genes or arranged as a spread of additional copies [2].

Based on the supposed homogeneity among 5S rDNA repeats, several studies propose that 5S rDNA are subject to concerted evolution [3-5], where duplicated gene family members evolve as a single unit that undergoes a high degree of homogenization (as a unit in concert). A combination of unequal exchange and gene conversion within and between the same chromosome loci have been suggested to explain how such evolution can occur "in concert" [6,7]. A key difference between theses mechanisms is that gene conversion maintains the copy number of a gene, whereas unequal crossing over may increase or decrease the gene copy number from generation to generation.

However, the diverse molecular features exhibited by the 5S genes and spacers bring into question the assumption that there is concerted evolution in 5S rDNA. First of all, the majority of the findings on concerted evolution of rDNA were based on the major ribosomal 18S, 28S and 5.8S units, which differ from 5S rDNA in number of repeats, genomic organization and transcriptional machinery. Not all mechanisms that affect major ribosomal genes may act on 5S rDNA arrays. Moreover, molecular analyses have demonstrated the existence of remarkable 5S rDNA variants [8-13] within individuals and species of plants [14,15], fungi [16,17] and animals [18-21]. Generally, these 5S rDNA variants correspond to paralogs copies that are clustered or dispersed in the genome. In several vertebrate groups, the main difference between 5S rDNA variants involves the length of the NTS, and are mostly due to single mutations or indels, whereas the transcribed regions of 5S rRNA are not divergent. Conversely, in studies of marine and freshwater fish [22-28], including members of the elasmobranch group such as sharks and rays [29-31], significant variation has been found in spacer sequences and even in the 5S rRNA genes. An extensive analysis of nucleotide sequences and chromosomal *in situ *hybridization, also in fish, demonstrated that such variant forms correspond to two classes of 5S rDNA repeats, each organized separately in the genome [see 30, for review]. Two classes of 5S rDNA were also observed in *Xenopus*; the first was expressed in somatic cells and the second, which was derived from the somatic type by gene duplication, was expressed in oocytes [21]. Together, these findings suggest that, aside from the mechanisms of the classical Dover-Arnheim model of concerted evolution, additional mechanisms are likely involved in the evolution of 5S rDNA.

Based on the evidence presented above, 5S rDNA families also have been proposed to evolve according to an evolutionary process known as birth-and-death [16,32-34]. In the birth-and-death model of evolution, new genes are created by repeated gene duplication at different genomic locations, and some of the duplicated genes are maintained in the genome for a long time, while others are deleted or become nonfunctional. On the other hand, homogeneity is maintained by the effects of strong purifying selection, and as a result, the DNA sequence of different members of the same gene family can be very different, both within and between species [33]. Consequently, high levels of intragenomic repeat variation are expected in the 5S rDNA repeats that evolve through birth-and-death process, leading to the accumulation of numerous 5S ribosomal gene and spacer variants [35]. To distinguish between concerted evolution and evolution by gene birth-and-death, knowledge about the level of repeat variation and the phylogenetic patterns between species is critical.

Studies of the structure and organization of 5S rDNA in the genome of chondrichthyans, a long-lived, well-adapted vertebrate group, are limited to a few marine families such as the Rajidae [29] and Carcharhinidae [30-32]. Similar to bony fishes, the genomes of sharks and rays also seems to harbor a dual 5S rDNA system [29], although additional variant copies have been detected in other species [36]. Furthermore, a distinctive 5S rRNA gene class shared only by elasmobranch species [30] suggests a group-specific evolutionary history of 5S rDNA, making these organisms of special interest for deciphering the genomic architecture of multigene families.

In the present paper, we investigated the genomic organization of 5S rDNA tandem repeats in members of the Potamotrygonidae family, who comprise the only group of rays that is totally restricted to freshwater systems [37,38]. These rays belong to the Myliobatiformes order as well, a large group of predominantly marine elasmobranches [39]. In our discussion, we focus on a model for the ongoing evolution of the 5S rRNA genes among elasmobranches. We also performed a comparative genomic analysis of 5S rRNA genes from several fish orders, as well as from other, unrelated vertebrates, in an attempt to measure the contribution of genomic events to the diversification of the 5S rRNA multigene family in long-term evolution.

Our results identified two types of 5S rDNA tandem repeats in stingrays, as was previously observed in teleosts and other elasmobranches. Nucleotide polymorphisms in the 5S rDNA sequences were also valuable as molecular markers to distinguish different genera and species of Potamotrygonidae stingrays. Finally, the large vertebrate dataset of 5S rDNA sequences examined support the idea that this multigene family evolves in the fish genomes according to a mechanism integrating both birth-and-death and concerted evolution.

## Results

### 5S rDNA organization in Potamotrygonidae stingrays

**Figure 1:**
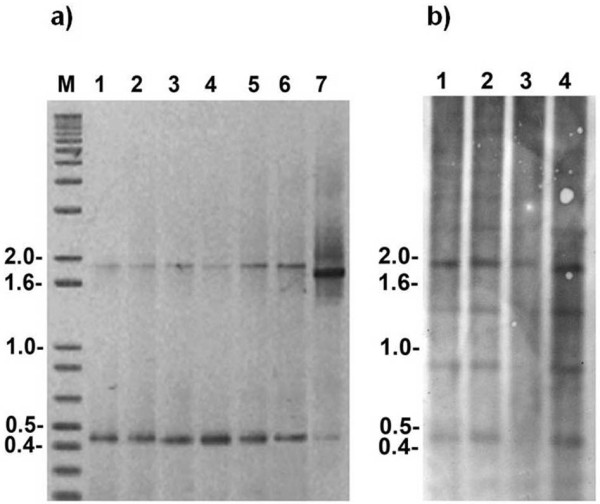
**PCR products and Southern blot probed to 5S rDNA of stingrays**. (a) PCR amplicons of 5S rDNA repeats from freshwater stingrays submitted to garose gel electrophoresis. 1-3, *Potamotrygon motoro*; 4-6, *Potamotrygon falkneri*; 7, *Paratrygon aiereba*. (b) Southern blot hybridization of *Hind*III digested genomic DNA of *P. falkneri *(1), *P. motoro *(2 and 3) and *P. aiereba *(4) probed with 5S rDNA class I of *P. motoro*. Bands of distinct molecular weight correspond to monomeric, dimeric, trimeric and tetrameric units, indicating sequence variants lacking the *Hind*III restriction site or undigested products. M, molecular marker with base pair sizes showed on the left.

Electrophoresis of PCR products from the 5S rDNA of Potamotrygonidae stingrays on ethidium bromide stained gels revealed the existence of two fragments of different sizes. A shorter fragment of ~450 bp in length was common to the three species, while larger fragments of ~1,800 bp in length were found in the congeners *P. motoro *and *P. falkneri*, and of ~1,700 bp in length in *P. aiereba *(Figure 1). Using BLASTn, sequences of several positive clones were confirmed to be 5S rDNA repeat units, each consisting of a 5S rRNA gene (120 bp) and an adjacent NTS of highly variable length (Table 1) (Genbank accession numbers JF92309-JF92336). We named the shorter and the larger fragments as 5S rDNA class I and 5S rDNA class II, respectively. The coding regions and nontranscribed spacers of the 5S rDNA class I were completely sequenced, (i.e., all 450 bp were recovered). However, from 5S rDNA class II clones, we were able to obtain the sequence of the entire coding region but only a partial segment of the NTS, for a total of 650 bp. After removing the non-informative primer annealing regions, we analyzed a 77 bp segment of the 5S rRNA gene (see additional file 1: Final alignment of nucleotide sequences encompassing the class I and class II 5S rRNA genes from the three Potamotrygonidae stingrays), plus a NTS segment of variable length in several clones from three individuals of each species (Table 1, see additional file 2: NTS class I and NTS class II nucleotide sequences from the stingray species included in this study), from both 5S rDNA classes. It is worth noting that the 77 bp segment we analyzed completely covers the internal control regions (ICRs), which are considered key regions of 5S genes due to their active role as transcriptional promoters. The A box is a general ICR sequence for RNA polymerase III. The intermediate element (IE) and the C box are specific to 5S rRNA transcription and work as binding sites for the transcription factor TFIIIA [40].

**Table 1:** Number of clones (NC), size (SL) and genetic distance (GD) of 5S rDNA units in Potamotrygonidae stingrays

	5S rDNA classe I			5S rDNA classe II		
	
	NC	SL	GD	NC	SL	GD
**5S rRNA gene**						
*P. falkneri*	06	77	0.012	03	77	0.018
*P. motoro*	06	77	0.040	06	77	0.000
*P. aiereba*	02	77	0.000	07	77	0.029
Overall average	14	77	0.017	16	77	0.016

**NTS**						
*P. falkneri*	06	360-361	0.009	04	592-594	0.023
*P. motoro*	06	360-361	0.003	06	586-604	0.029
*P. aiereba*	02	369	0.014	06	565-609	0.035
Overall average	14	360-369	0.016	16	565-609	0.078

NC, number of clones; SL, Sequence length; GD, Genetic distance

**Figure 2:**
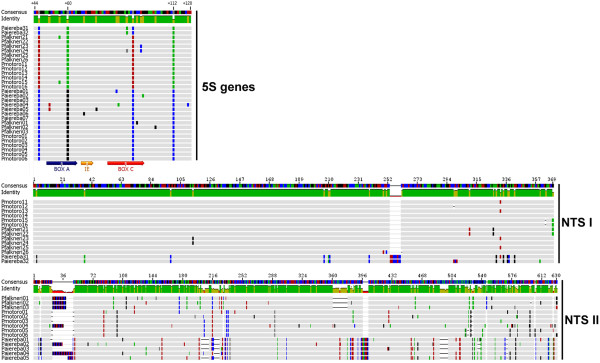
**Consensus and aligned sequences of the 5S genes, short spacers (NTS I) and long spacers (NTS II) found in freshwater stingrays**. Nucleotides color legend: Adenine = green; Cytosine = blue; Guanine = black; Thymine = red. Grey bars denote identical bases; dashes represent gaps. In the coding sequences, colored arrows indicate internal control regions (Box A, intermediate element (IE), and Box C).

The subsequent alignment of Potamotrigonidae 5S rRNA genes allowed us to identify two types of sequences that are somewhat distinct. These stingray sequences probably correspond to functional genes because they encompass the internal control regions (ICR) within the coding segment (i.e., box A, internal element and box C) and the poly-T motif at the end of the coding region. We also found the 5S genes to be GC-rich (53.3% to the class I and 58.4 to the class II). Only two nucleotide polymorphisms, at positions 60 and 112 (a G-A transition, and a C-A transversion, respectively), characterized both sequences in the two classes from the three species (Figure 2, see additional file 1: Final alignment of nucleotide sequences encompassing the class I and class II 5S rRNA genes from the three Potamotrygonidae stingrays).

Numerous dispersed non-parsimony informative mutations were also found in each 5S gene class. The genetic variation was lower when comparing 5S gene classes in *Paratrygon*. The overall genetic distance of coding sequences among the three species was also low, with a mean of 0.17 and 0.16 for class I and class II, respectively (Tables 1 and 2). Among the ICRs, we detected extensive nucleotide variation within the A and C boxes and a unique polymorphism within the internal element (Figure 2, Table 2, see additional file 1: Final alignment of nucleotide sequences encompassing the class I and class II 5S rRNA genes from the three Potamotrygonidae stingrays).

**Table 2:** Polymorphism (π, bold values), divergence (K, lower diagonal) and fixed differences (upper diagonal) in 5S rRNA genes among stingrays

	*P. aireba*	*P. falkneri*	*P. motoro*
*P. aiereba*	**0.03849 ± 0.007**	0	0
*P. falkneri*	0.05405 ± 0.002	**0.04195 ± 0.009**	0
*P. motoro*	0.04268 ± 0.001	0.03530 ± 0.001	**0.03164 ± 0.004**

Southern blot hybridization experiments confirmed the existence of two 5S rDNA classes in Potamotrygonidae composed of tandem repeats of ~450 bp and ~1,700 to 1,800 bp. This finding is further supported by the PCR products and the sequencing data obtained (Figure 1).

In contrast to the way the 5S rRNA genes within and between 5S rDNA classes were conserved or only moderately variable, we discovered two very distinct NTS types in the genomes of the three stingrays. Furthermore, data mining showed that NTS sequences isolated from Potamotrygonidae do not match any nucleotide sequence currently in the NCBI database.

We detected a high degree of sequence-length polymorphism within the two NTS classes. Class I spacers had 12 nucleotides substitutions plus six extra bases (a TCC repeat), which distinguishes *P. aiereba *from the other *Potamotrygon *species, whereas the two congeners had no species-specific nucleotides (see additional file 2: NTS class I and NTS class II nucleotide sequences from the stingray species included in this study). Despite partial sequencing, the large NTS class II had several interspecific polymorphisms, which allowed the congeners of *P. motoro *and *P. falkneri *spacer sequences to be discriminated (see additional file 2: NTS class I and NTS class II nucleotide sequences from the stingray species included in this study). Both NTS classes had a high GC content (class I = 56.3% and class II = 59.7%), comparable to the 5S gene. This was unexpected because GC-rich regions are predominantly found inside coding sequences. Table 1 shows the analysis of several intraspecific parameters. We detected low similarity between orthologs class II NTS regions, which is in contrast to how highly conserved between *Potamotrygon *and *Paratrygon *genera the class I regions are. The highly polymorphic class II spacers contrast with the homogenous spacers of class I (Table 1, Figure 2). Indels and nucleotide substitutions further increase genetic distance within NTS type II sequence (Figure 2, see additional file 2: NTS class I and NTS class II nucleotide sequences from the stingray species included in this study). The estimates of average evolutionary divergence between all NTS sequence pairs were much higher in the larger spacers (NTS I: 0.016 ± 0.003; NTS II: 0.081 ± 0.009).

We conducted intra- and interspecific analyses between the two paralogs NTS classes independently. Intraspecific nucleotide diversity in stingrays was always less than 0.03; however, the percentage of polymorphisms was consistently beneath 7%. These data suggest the occurrence of several variants that are different by only one or a few nucleotides. Furthermore, simple sequence repeats (SSRs) that represent highly mutable DNA sequences were detected in the NTS II class and significantly contributed to the observed intra- and interspecies nucleotide variation (Figure 2). In a detailed analysis of the NTS II, we identified several tri- and dinucleotide SSRs composed of TGC and AC/GC motifs, respectively, which may be responsible for the high number polymorphisms observed. Finally, indel blocks outside the SSRs ranging from 7 to 18 bp were found to exist either in *Potamotrygon *or in *Paratrygon *sequences (see additional file 2: NTS class I and NTS class II nucleotide sequences from the stingray species included in this study).

### Phylogenetic inferences based on the 5S genes and NTSs of Potamotrygonidae stingrays

**Figure 3:**
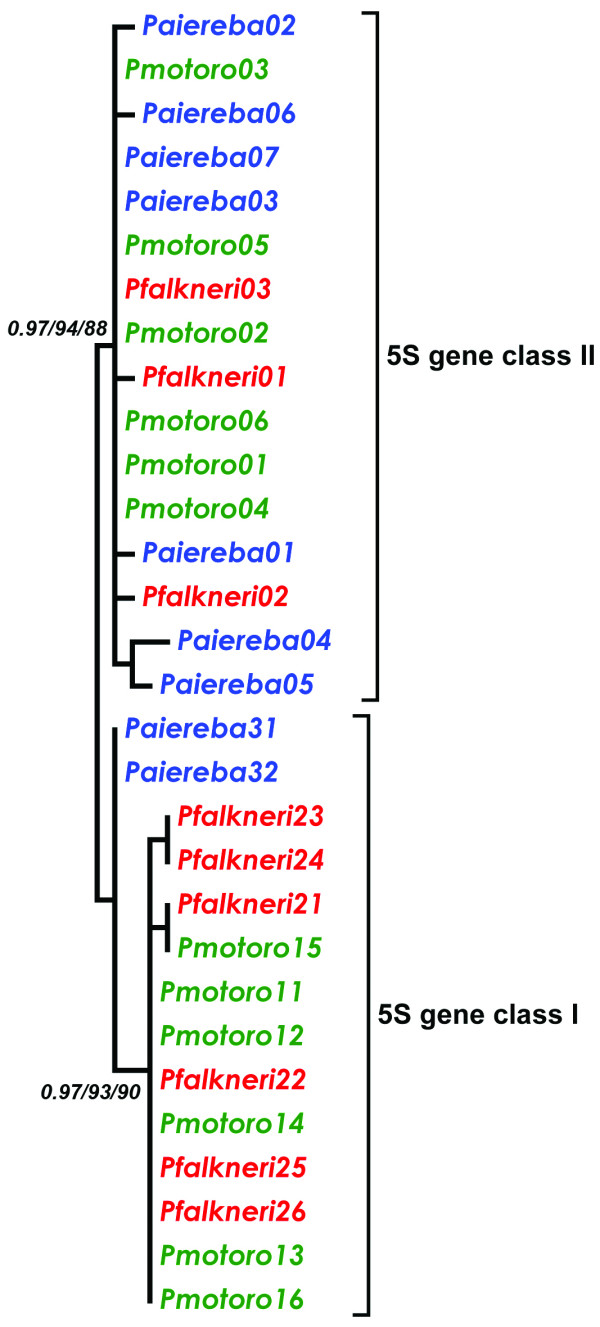
**Phylogenetic consensus tree based on 5S rRNA gene sequences of the Potamotrygonidae species *Potamotrygon motoro, P. falkneri *and *Paratrygon aireba***. Statistical support for BI/ML/MP analyses are indicated by triplets of numbers and were obtained by posterior probabilities (BI) and bootstrap (ML and MP) methods after 1000 replicates.

**Figure 4:**
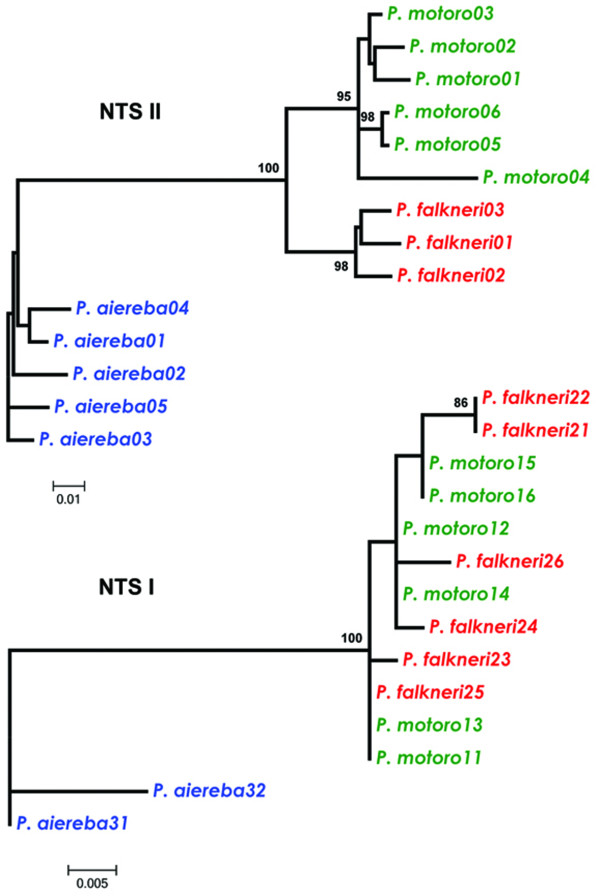
**Maximum-likelihood trees of the two types of NTS sequences, NTS class I and NTS class II**. Values represent nodes recovery percentages after 1000 bootstrap replicates. Bootstrap values under 50% are omitted.

We performed independent phylogenetic analyses of the 5S genes and the NTS sequences, between and within each Potamotrygonidae species (Figures 3 and 4). Modeltest 3.6 [41] determined that the Transitional Model with Equal Frequencies (TIMef) was the best model for the evolution of 5S genes; however, for the NTS, Felsentein 81 (F81) was the best fitting model. Both models incorporate rate variation among sites (+G).

Applying these parameters to all examined specimens supports the existence of two classes of 5S rDNA that are well separated into distinct clades in the phylogenetic trees for the 5S genes and the NTS (Figures 3 and 4, respectively). Phylogenetic data showed that variability in the NTS is responsible for the separation of 5S rDNA into two classes. However, the 5S rRNA genes also harbor polymorphisms that differentiate them into two classes (classes I and II), as illustrated by the high probability values for the BI, ML and MP trees (Figure 3). Analyzing the relationships in the 5S rRNA gene tree, we determined that the class I genes from *P. aiereba *branched from the *Potamotrygon *species class I genes due to two transitions at positions 46 and 92 (Figure 2). In the corresponding positions, *P. aiereba *carries the same two nucleotides shared by its class II genes, as well as by the class II genes of all *Potamotrygon *individuals (see additional File 1: Final alignment of the nucleotide sequences of the 5S rRNA class II and class I genes from three Potamotrygonidae stingrays). The class II genes from all individuals do not cluster according to species boundaries, with intermingling between classes (Figure 3). This finding indicates that most of the phylogenetic relationships seen are likely not significant, and therefore, only the differentiation between variant copies of class I and II genes is well supported.

When evaluating NTS data, the *P. aiereba *spacers always cluster in a separate branch from those of the *Potamotrygon *species. The ML tree of NTS class I sequence clearly identified two clades of sequences (Figure 4). One clade was comprised of the *P. aiereba *NTS I sequences, which branched out according to species boundaries. The other clade was composed of intermingled sequences from the two *Potamotrygon *species. In contrast, the comparison of NTS class II sequences produced a tree that was more informative in distinguishing the relationship between the three species (Figure 4), as all branches obtained very high statistical support.

### Phylogenetic analysis of vertebrate 5S genes

**Figure 5:**
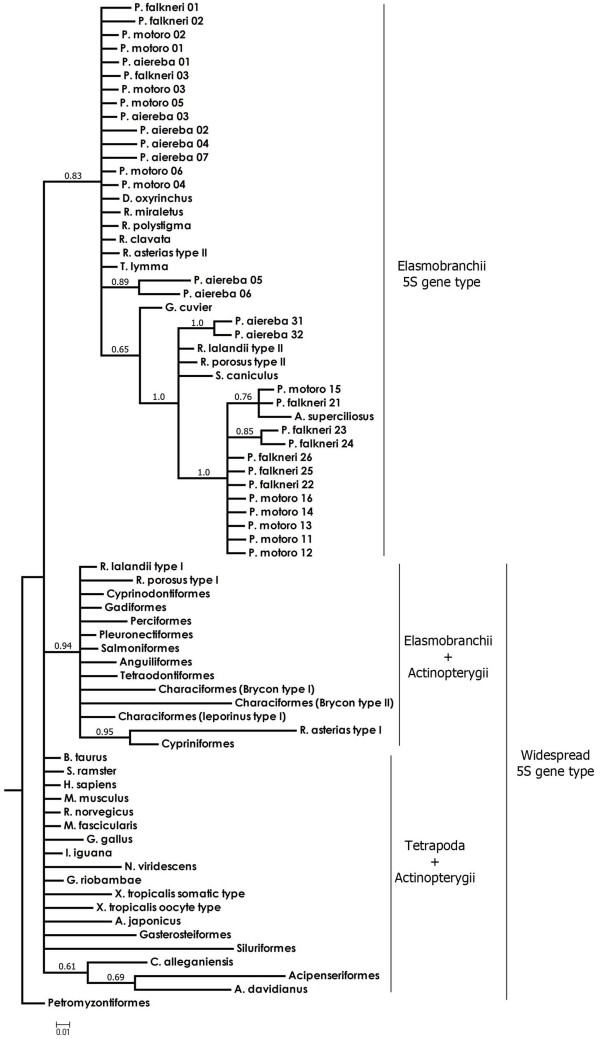
**Bayesian phylogenetic tree for 100 vertebrate species based on 5S gene sequences using lampreys (Agnatha) as outgroup of all the other (Gnathostomata)**. Values represent nodes posterior probability support recovered in the Bayesian analysis.

We verified several divergent vertebrate clades using a Bayesian tree, and by examining the alignment of 5S coding sequences (Figure 5, see additional file 3: Alignment of 5S rRNA gene sequences from several vertebrates). The most basal division we could detect was between jawless fish such as lampreys (Agnathans) and jawed vertebrates (Gnathostomata). A consistent phylogenetic signal (83% posterior probability) supports a clade containing only the Elasmobranchii, except for the class II sequences from *Raja asterias *and the class I sequences from *Rhizoprionodon lalandii *and *R. porosus*, which clustered in a separate branch with Teleostei. Moreover, the "elasmobranch clade" splits into two subclades comprising the shark and Potamotrygonidae class I genes (65% posterior probability) as a sister branch of the Rajidae and Potamotrygonidae class II genes. Therefore, both 5S gene variants in Potamotrygonidae stingrays were placed in the elasmobranch clade, joining either sharks or rays (Figure 5). In another clade, the 5S genes of Ray-finned fishes, represented by Chondrostei (Acipenseriformes) and two Teleostei (Gasterosteiformes and Siluriformes), intermingled with Tetrapoda (Reptilian, Amphibian, Avian and Mammal). The remaining Teleostei fish 5S gene sequences clustered into a single clade with the 5S gene class genes from *Raja asterias, R. lalandii *and *R. porosus *(Figure 5).

## Discussion

### Molecular organization, evolution and biological significance of the two 5S rDNA arrays in stingrays

The cause of the widespread appearance of distinct classes of 5S rDNA repeats in the genomes of vertebrate species is unknown. In fishes, distinct classes of 5S rDNA have already been found in several Actinopterygii and Elasmobranchii. Usually, the variation in 5S rDNA is related to the high polymorphism in the NTS regions, whereas the 5S coding region remained unchanged [25,42]. However, in *Merlucius *fishes [28], Rajidae rays [29] and Carcharhinidae sharks [30,31], 5S genes were found to be quite divergent between variant classes and still functional.

In this paper, we identified the characteristic 5S rDNA dual system in the three freshwater stingray genomes (*P. motoro, P. falkneri *and *P. aiereba*) as determined by PCR, sequencing and Southern blotting. Interspecific examination of the two classes of 5S rDNA revealed notable dissimilarities between NTS I and II classes, in agreement with previously published data [24-26]. By contrast, we found a low quantity of intraspecific variation within each NTS class, where sequence homogenization was more intense. The notable genetic distances found in the between-classes comparison (NTS I sequences had 0.016 and NTS II had 0.078 of overall genetic distance) suggest that both NTS classes are under distinct genomic pressures. It is interesting to note that the shorter NTS (NTS I) has been extensively homogenized when compared to the longer NTS II. The low level of variation in the NTS I could be related to selective pressure against major changes that could disrupt essential regulatory elements present on this NTS. It has been argued that a minimum NTS size is necessary for the maintenance of 5S rDNA repeats in the genome [43] because the NTSs could contain DNA elements involved in the regulation of 5S rRNA gene expression [44,45]. On the other hand, the variable levels of homogenization of NTSs I and II (and consequently of both 5S rDNA classes) could be explained by the clustering of each class in distinct genomic environments under constraint from different evolutionary forces.

**Figure 6:**
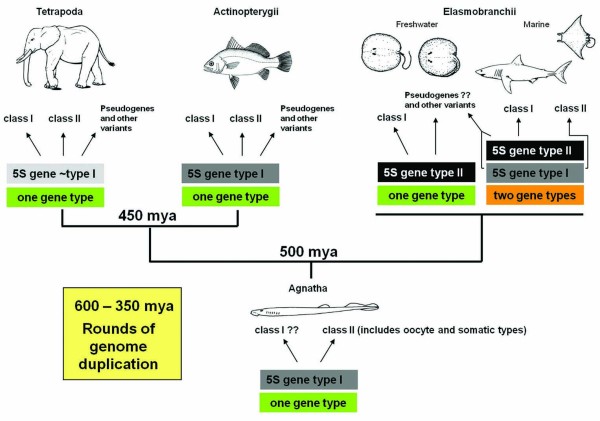
**Proposed evolutionary history for the 5S rRNA genes among vertebrates**. Rounds of genome duplication times indicated according to [73-75].

In sharks, the ancestral 5S class I genes derive from different duplicated genes that originated before the separation of the Agnathans and the Gnathostomans species [30]. In contrast, although there was a slight variation in the 5S rRNA genes of stingrays, they still corresponded to a single gene type, noticeably, the elasmobranch type. Stingrays do not appear to carry the ancestral 5S gene class I in their genomes, indicating that it may have been lost in the course of evolution due to a lack of functional constraint. Within the Elasmobranchii group, stingrays can be considered a modern division that irradiated approximately 19 mya from a marine ray ancestor [46,47], 180 mya after the separation of Batoidea (rays) and neoselachians (sharks) in the basal Jurassic or late Triassic periods [48]. Marine stingrays studied thus far have an ancestral class I gene; therefore, the loss of this gene variant in freshwater stingrays may have occurred later, during the evolutionary transitions from marine to freshwater habitats. Bearing in mind that variant copies of 5S rRNA multigene families are commonly found dispersed across vertebrate genomes, the birth-and-death of genes following mutational events perhaps led to the loss of the ancestral class I gene (Figure 6). Given that new gene clusters arise mostly by chance, the initial evolution of classes within multigene families may be casual. Moreover, considering the feasibility of transposition of the 5S rRNA genes [49,50], new genes could arise by new fixation of unfastened short number copies from pre-existent tandem arrays (e.g., the class II arrays). Afterward, duplications would spread, and these genes would form a new cluster, as can be observed in other multigene families [51,52]. Thus, functional or even non-functional sequences could be kept and homogenized by gene conversion and unequal crossing over via the process of concerted evolution, leading to the current set of observed variant gene classes. Similarly, non-functional variant copies of 5S rDNA repeats could also spread and give rise to several new clusters in the genome, as observed in the *Hoplias malabaricus *fish [53].

It still remains unclear whether the two 5S rDNA classes of vertebrates are both related to the canonical role of ribosomal RNAs or represent a distinct biological feature. The occurrence of 5S genes that are differentially regulated in somatic and oocyte cells [21] has been explored as the reason for the dual 5S rDNA pattern reported in fish. However, neither the somatic nor the oocyte types are correlated with the divergent 5S rDNA classes detected in diverse fish groups [30] as well as the Potamotrygonidae stingrays.

Among fishes, chromosomal data support the idea that the divergence of different classes of 5S rDNA constitutes a widespread form of organization, and is related to their presence in different chromosomal regions [25]. Nevertheless, even divergent 5S rDNA types that differ profoundly in their spacer sequences and genomic environments may be situated on the same chromosome [42,54]. Differently sized units may be arranged in composite tandem arrays, as is seen in several species of plants [55], such as in *Vitis vinifera*, where three different 5S rDNA units coexist within the same tandem array [56]. The same arrangement has been observed in six Mugilidae fishes of the genera *Liza *and *Chelon*, where two types of 5S rDNA repeat units were characterized by an intermixed arrangement within a single chromosome locus [27].

In the Potamotrygonidae, although we do not have direct evidence of how the two 5S rDNA tandem arrays are clustered, we have some evidence that they are disconnected. First, we did not observe any evidence of an intermixed arrangement of 5S rDNA classes I and II from the PCR or Southern blot results. If 5S rDNA type I and II were intermixed, we would obtain different band patterns, rather than the classical tandem repeat patterns observed. Furthermore, the intraspecific comparison of different classes revealed a high dissimilarity between them, which suggests they occupy distinct chromosome locations.

Data accumulated to date demonstrates that the presence of two distinct classes of 5S rDNA arrays in elasmobranches and teleost fishes is a general trend (Figure 6). Such variation may be a consequence of ancient diversification of 5S rDNA repeat types and its fixation in the main lineages of vertebrates. The intense genomic dynamism that seems to guide the evolution of tandem repeat elements may have generated the divergent copies of 5S rDNA observed. Some authors [57] speculate that the newly generated duplicate genes or gene families may evolve to interact with other existing gene families and promote the adaptation of organisms to new environments. However, there are no substantial evidences for such a conclusive statement, and, therefore, the major biological reason for such a dual pattern of 5S rDNA organization remains unknown.

### 5S rDNA as phylogenetic and phylogeographic tool and its utility for the molecular identification of stingrays

The 5S rDNA polymorphisms were efficient markers for the discrimination of genera and species of potamotrygonids. The two 5S rDNA classes characterized in *P. motoro, P. falkneri *and *P. aiereba *represent paralogs and should be treated as two different nuclear markers. Nucleotide-level and sequence length variation generate distinct profiles by PCR (Figure 1) and in phylogenetic trees (Figure 4), which is useful for distinguishing between *Potamotrygon *and *Paratrygon *species. Unfortunately, we were not able to identify the three species using PCR alone, as can be done for a few shark species [36].

Although the NTS I class sequences were highly uniform within the *Potamotrygon *genus, a few consistent polymorphisms were enough to detach them from *Paratrygon*. By contrast, intraspecific nucleotide variability was 5-fold higher in NTS II class than in NTS I; therefore, NTS I can be used to discriminate *P. aiereba *from the *Potamotrygon *spp. Alternatively, NTS II discriminates all potamotrygonid species and represents an excellent marker to access species identification within this group.

5S rDNA sequences and the NTS segments in particular have been successfully applied to the identification and inspection programs intended to assess the identity of species and hybrids [36,58,59], as well as in fish phylogeography [60] and phylogenetic inference studies [29,61]. In Potamotrygonidae, short and long repeats may also correspond to informative markers at the population level and may be used in combination with mitochondrial markers in a more consistent dual analysis. Further independent characterization of each 5S rDNA class in stingray populations from distinct areas would provide a better basis for phylogeographic studies. Hence, the development of new genetic markers is a welcome contribution to the study of phylogeny, phylogeography and identification in potamotrygonids species.

### A mixed model for the evolution of 5S rDNA

Several mechanisms are believed to act in the evolution of multigene families and duplicated sequences, driving their accumulation, divergence or even deletion from the genome. The model of concerted evolution was originally thought to apply to gene families that are responsible for producing a large quantity of the same gene product, as in the case of rRNA genes [62].

Since the concerted evolution theory explains the observed lack of genetic variability among rRNA gene copies in many different species, it became a universal accepted as the unique mode of evolution of rRNA multigene families [63-65].

Although concerted evolution explains sequence homogenization among members of a repeated gene family, a pair of paralog gene sequences can diverge fast enough to escape gene conversion [66]. When this happens, the rate of homogenization may be too low to prevent significant levels of intraspecific rDNA polymorphisms. If this is the case, the production and maintenance of a large quantity of the same gene product can also be achieved by strong purifying selection, without concerted evolution [57]. When concerted evolution takes place, it will homogenize the gene copies that are arrayed in the same cluster, and this cluster can differ significantly from paralogs copies of a second cluster. Thus, new genes are created by gene duplication, and some duplicated genes are maintained in the genome for a long time, but other genes are deleted or become nonfunctional (e.g., pseudogenes) through deleterious mutations.

Most studies in teleost fish have shown that a majority of 5S rRNA multigene families evolve in a rigorously concerted fashion. However, the presence of several 5S rDNA variants has been reported in such diverse taxa as molluscs [8,12,13,67], echinoderms [68] and arthropods [10]. Among fishes, highly variable paralogs have been clearly documented in the 5S rDNA regions in species of the *Leporinus *genus [25], in the Nile tilapia *Oreochromis niloticus *[42], in *Merlucius *species [28], distinct Actinopterygii fish orders [9,24] and in marine Elasmobranchii [29,30]. Our data advocate the hypothesis that independent mechanisms guide the evolution of distinct 5S rDNA classes in the genome of stingrays, which are likely maintained by both concerted and birth-and-death evolution, as was reported for bitterling (Cyprinidae) fish [69].

In fact, a variety of studies have shown that rDNA and other multigene families can evolve through distinct mechanisms, leading to evolutionary patterns other than concerted evolution. Highly conserved histone and ubiquitin gene families are well-defined examples of birth-and-death evolution [34,70-72]. For example, small gene families with strong purifying selection, such as the heat shock protein [73] and amylase gene families [74], evolve through a mix of evolutionary processes.

The presence of pseudogenes in a multigene family strongly suggests that the family evolves by a birth-and-death process [16]. There are numerous reports of rRNA pseudogenes where the coding regions do not have functional constraints [10,75-77]. In fish, diverse studies have reported the presence of 5S rDNA pseudogenes [24,28,42,69]. In Potamotrygonidae, no evidence of pseudogenes was found, most likely because both primers anneal in the 5S rRNA gene, and the presence of mutations in the gene would have considerably reduced the likelihood that pseudogenes would have been amplified, cloned, and sequenced [35]. A larger genomic survey in the future could be useful to detect pseudogenes in stingrays.

Under the concerted evolution model, genes cluster according to species; however, they do not under the birth-and-death model, except in cases of recent gene duplication [16]. The variant sequences of 5S rDNA from the three different stingray species clustered according to classes but not to species. These findings indicate that birth-and-death processes have been active throughout Potamotrygonidae 5S rDNA evolution. Conversely, nucleotide diversity values calculated for species within each clade were relatively small, implying that homogenizing forces diminished sequence divergence locally within each separate cluster of 5S rDNA variants in each species. Such a pattern fit in a mixture of concerted and birth-and-death evolution, where distinct arrays tend to accumulate large amounts of variation that is kept or lost due to purifying selection. However, paralogs copies in the genome, representing distinct 5S rDNA arrays, appear to undergo array-specific concerted evolution rather than a single homogenization mechanism common to all arrays.

To understand the evolutionary dynamics of 5S rRNA genes, we carried out a large cross-species survey. Figure 5 shows the phylogenetic tree of 5S genes from major vertebrate lineages. The analyses performed showed a between-species clustering of 5S ribosomal DNA variants. As expected from previous studies [30], despite being distantly related, several organisms, including sharks, frogs, mice and humans, share the widespread ancestral forms of the 5S genes. Furthermore, the majority of vertebrate lineages contain variant 5S rDNA copies, which have differentiated from the original genes by duplication and deletion events.

Studies suggesting the occurrence of birth-and-death on rDNA arrays have been predominantly conducted using lower eukaryotes, such as studies of the 18S rDNA in Apicomplexans [78], fungi [16], plants [79] and invertebrates [35], who theoretically differ from higher eukaryotes in genome dynamics. Thus, our broad survey in vertebrates can shed light on the evolution of rDNA arrays.

In *Potamotrygon *and *Paratrygon *species, as well as in sharks and teleost fish, we found several 5S rDNA variants within clusters, even in the 5S rRNA gene. Comparisons between variants demonstrated a lack of homogenization in the Elasmobranchii and Teleostei, whereas homogenizing mechanisms appeared to be active within each variant in each species. These new variants emerged sporadically during fish evolution in the main vertebrate lineages and likely originated during ancient rounds of genome duplications [80-82] acting on polymorphic ancestral 5S rDNA arrays. This hypothesis is supported by phylogenetic analyses that revealed a between-species clustering of Potamotrygonidae 5S rDNA variants, which was also observed in the rDNA sequences of sharks, marine rays and several teleost fish 5S [29,30,69,83]. Thus, the emergence of new variants and their within-variant homogenization supports the idea that both concerted and birth-and-death evolution are responsible for the extant variation of this multigene family in fish genomes. Recently, similar results were found in invertebrates [13], suggesting that the long-term evolution of 5S rDNA is most likely mediated by a mixed mechanism in which the generation of genetic diversity is achieved through birth-and-death. This process is then followed by the local homogenization of the paralogs units, which most likely occurs after their physical movement to independent chromosomal locations.

## Conclusions

A complex combination of duplications, insertions, deletions, and general genome rearrangements has likely been involved in the evolution of the 5S rRNA gene family in vertebrates. The present work reveals that in fishes, different classes of 5S rDNA are organized in distinct clusters that arose from duplications and are kept or lost by purifying selection under birth-and-death evolution. Simultaneously, unequal crossing over and gene conversion homogenize tandemly arrayed gene copies in each cluster, leading to the observed pattern of concerted evolution. Therefore, we concluded that 5S rDNA in fish genomes appears to evolve according to the mixed effects of concerted and birth-and-death evolution.

## Methods

### Sampling, cloning and sequencing protocols

Fresh samples of ten *Potamotrygon falkneri*, twelve *Potamotrygon motoro *(collected in Rio Paraná, Três Lagoas/MS, Guaíra/PR and Foz do Iguaçu/PR, Brazil) and one *Paratrygon aiereba *specimen (collected in Rio Purus, Porto Velho/RO, Brazil) were subjected to genetic analysis. DNA was isolated from fins [84], and PCR amplifications of the 5S rDNA were performed using the Cart5S1f (5'-CAC GCC CGA TCC CGT CCG ATC-3') and Cart5S1r (5'-CAG GCT AGT ATG GCC ATA GGC-3') primers. These oligonucleotides were designed by [30] based on the 5S rRNA gene sequence of the elasmobranches *Scyliorhinus caniculus *(GenBank entry M24954) [85] and *Taeniura lymma *(GenBank entry AY278251) [86].

PCR amplifications were performed using 150 pmol of each primer, 20-80 ng of genomic template DNA, 1x Taq buffer, 200 μM of dNTPs, and 1 U of Taq polymerase (Invitrogen) in a final reaction volume of 25 μl. The cycling times were: 5 min at 94°C; 35 cycles of 1 min at 95°C (denaturation), 30 s at 55°C (annealing) and 45 s at 72°C (elongation); and a final 5 min extension at 72°C. A negative control was always included to determine if any contamination occurred. The PCR products were resolved in 1% agarose gels and compared with a standard DNA marker (1Kb Plus Ladder - Invitrogen). Fragments were visualized after ethidium bromide staining, and the gel image was recovered using the EDAS program (Electrophoresis Documentation and Analysis System 120 - Kodak Digital Science 1D).

The PCR products were cloned into pGEM-T plasmids (Promega) and were used to transform DH5á *Escherichia coli *competent cells. Positive recombinant clones were recovered and stored in 75% glycerol at -80°C. The positive clones were sequenced on an ABI Prism 3100 automatic DNA sequencer (Applied Biosystems) with a Dynamic Terminator Cycle Sequencing kit (Applied Biosystems) following the manufacturer's instructions.

### Phylogenetic analysis

After remove vectors and primers sequences, we subjected nucleic acid sequences to BLASTn searches at the National Center for Biotechnology Information website (http://www.ncbi.nlm.nih.gov/blast). Next, sequence alignments were performed using MUSCLE [87], and consensus sequences were produced manually using BioEdit software [88].

Phylogenetic trees were generated by Bayesian Inference (BI), Maximum Likelihood (ML) and Maximum Parsimony (MP) methods employing the best fitting model of evolution, which was previously selected for each dataset following the Akaike Information Criterion (AIC) obtained with Modeltest 3.6 [41]. Maximim Likelihood trees were constructed with the PhyML program [89,90] using a website version (http://hcv.lanl.gov/content/sequence/PHYML/interface.html). Gamma shape parameters and the proportion of non-variant sites were estimated by maximum likelihood from a neighbor-joining tree (BIONJ). Maximum Parsimony trees were recovered in PAUP v4.0 [91] applying a branch-and-bound search and treating insertions/deletions as missing data. The support for individual nodes in ML and MP trees were assessed by bootstrap resampling [92] using 1,000 replicates with random additions and TBR branch swapping. Bayesian Inference trees [93] were generated via the estimation of posterior probabilities using MrBayes v.3.0 [94]. Two runs of four continuous-time Markov chains were performed simultaneously for each dataset using default heating and sampling every 100 cycles. Each run was 1,000,000 steps long, and the asymptote of the likelihood score was detected with the SUMP command.

Genetic distances over all sequence pairs were obtained in MEGA 4 [95], with bootstrap for 1,000 replicates. All positions containing gaps in the alignment and missing data were eliminated in pairwise sequence comparisons. Nucleotide diversity and divergence were calculated with DnaSP v5 [96].

5S rDNA sequences representing the most important live lineages of vertebrates were retrieved from GenBank/EMBL/DDJB (See additional file 4: Compilation of information regarding 5S rDNA nucleotide sequence in vertebrates) and were used in a comprehensive phylogenetic analysis. The majority-rule consensus (MRC) sequences of several Tetrapoda and Actinopterygii taxa were used to recover BI, ML and MP trees. Petromyzontifom lampreys were used as outgroup. Trees were visualized with the TreeExplorer program implemented in MEGA 4 [88].

### Southern blot hybridization

Around 10 μg of genomic DNA from *P. falknerii, P. motoro *and *P. aiereba *were completely digested with *Pst*I, *Hind*III, *Pvu*II and *Ssp*I endonucleases. These enzymes were selected based in their pattern of cut detected in the 5S rRNA gene sequences. The restriction products were subjected to 1% agarose gel electrophoresis and transferred to a Hybond-N^+ ^nylon membrane by capillary blotting [77]. DNA hybridization was performed using as probes the 5S rDNA sequences from *P. motoro *and *Hind*III digested DNA of the tree species. For the final labeling and detection steps, we employed the ECL-Direct Nucleic Acid Labeling and Detection System kit (GE Healthcare Biosciences), following the manufacturer's instructions.

## Authors' contributions

DP worked in the obtainment and analysis of nucleotide sequence data and drafted the manuscript. TSY and CSA helped in the DNA cloning and sequencing. CM designed and coordinated the study, and drafted and revised the manuscript. All authors read and approved the final manuscript.

## Supplementary Material

Additional file 1 **Final alignment of nucleotide sequences encompassing the class I and class II 5S rRNA genes from the three Potamotrygonidae stingrays**. Species are referred to as follows: Pfalkneri = *Potamotrygon falkneri*, Pmotoro = *P. motoro*, Paireba = *Paratrygon aiereba*. Dots represent sequence identity, gray shadowed nucleotides are indicative of distinctive sites between 5S genes found in the two 5S rDNA arrays. The internal control regions (A box, IE and C box) are highlighted in black. a, 5S rRNA genes class II; b, 5S rRNA genes class I. Click here for file

Additional file 2 **NTS class I and NTS class II nucleotide sequences from the stingray species included in this study**. a) Nucleotide sequence alignment of short NTS repetitions of class I; (b) partial nucleotide sequence alignments of long NTS repetitions of class II obtained from the stingrays genome. Species are referred to as follows: Pfalkneri = *Potamotrygon falkneri*, Pmotoro = *P. motoro*, Paireba = *Paratrygon aiereba*. Dots represent conserved nucleotides and hyphens report indels. The microrepetition TCCC expanded in the *P. aiereba *genome is indicated in gray shading. Dots represent conserved bases and hyphens report indels. Click here for file

Additional file 3 **Alignment of 5S rRNA gene sequences from several vertebrates**. The 5S gene of lampreys and diverse bony fish orders are majority-rule consensus (MRC) sequences obtained from data source listed in Additional file 1. (s) somatic type; (o) oocyte type. Sequences generated in the present study are underlined. Click here for file

Additional file 4 **Compilation of information regarding 5S rDNA nucleotide sequence in vertebrates**. 5S rDNA information of several vertebrate groups (except stingrays sequences) were retrieved from GenBank/EMBL/DDBJ and used in the present study. Click here for file

## References

[B1] Long EO Dawid IB Repeated genes in Eukaryotes Annu Rev Biochem 1980 49 727 764 10.1146/annurev.bi.49.070180.003455 6996571

[B2] Drouin G Moniz de Sá M The concerted evolution of 5S ribosomal genes linked to the repeat units of other multigene families Mol Biol Evol 1995 12 481 493 7739390 10.1093/oxfordjournals.molbev.a040223

[B3] Arnheim N Krystal M Schmickel R Wilson G Ryder O Zimmer E Molecular evidence for genetic exchanges among ribosomal genes on nonhomologous chromosomes in man and apes Proc Natl Acad Sci USA 1983 77 7323 7327 10.1073/pnas.77.12.7323 PMC350495 6261251

[B4] Dover G Molecular drive: a cohesive mode of species evolution Nature 1982 299 111 116 10.1038/299111a0 7110332

[B5] Arnheim N Nei M and Koehn RK Concerted evolution of multigene families Evolution of genes and proteins 1983 Sunderland: Sinauer 38 61

[B6] Dover GA Tautz D Conservation and divergence in multigene families: alternatives to selection and drift Phil Trans R Sot Lond B 1986 312 275 289 10.1098/rstb.1986.0007 2870522

[B7] Dover GA Molecular drive in multigene families: how biological novelties arise, spread and are assimilated Trend Genet 1986 2 161 165

[B8] Freire R Insua A Méndez J *Cerastoderma glaucum *5S ribosomal DNA: characterization of the repeat unit, divergence with respect to *Cerastoderma edule*, and PCR-RFLPs for the identification of both cockles Genome 2005 48 427 442 10.1139/g04-123 16121240

[B9] Robles F de la Herran R Ludwig A Rejon CR Rejon MR Garrido-Ramos MA Genomic organization and evolution of the 5S ribosomal DNA in the ancient fish sturgeon Genome 2005 48 18 28 10.1139/g04-077 15729393

[B10] Keller I Chintauan-Marquier IC Veltsos P Nichols RA Ribosomal DNA in the grasshopper *Podisma pedestris*: escape from concerted evolution Genetics 2006 174 863 874 10.1534/genetics.106.061341 16951064 PMC1602095

[B11] Sword GA Senior LB Gaskin JF Joern A Double trouble for grasshopper molecular systematics: intra-individual heterogeneity of both mitochondrial 12S-valine-16S and nuclear internal transcribed spacer ribosomal DNA sequences in *Hesperotettix viridis *(Orthoptera: Acrididae) Syst Entomol 2007 32 420 428 10.1111/j.1365-3113.2007.00385.x

[B12] López-Piñón MJ Freire R Insua A Méndez J Sequence characterization and phylogenetic analysis of the 5S ribosomal DNA in some scallops (Bivalvia: Pectinidae) Hereditas 2008 145 9 19 10.1111/j.0018-0661.2008.2034.x 18439229

[B13] Freire R Arias A Insua A Méndez J Eirín-López JM Evolutionary dynamics of the 5S rDNA gene family in the mussel Mytilus: mixed effects of birth-and-death and concerted evolution J Mol Evol 2010 70 413 426 10.1007/s00239-010-9341-3 20386892

[B14] Ganal MW Lapitan NLV Tanksley SD A molecular and cytogenetic survey of repeated DNA sequences in tomato (*Lycopersicon esculentum*) Mol Gen Genet 1988 213 262 268 10.1007/BF00339590

[B15] Nedi MS Rajagopal J Chauhan N Cronn R Lakshmikumaran M Length and sequence heterogeneity in 5S rDNA of *Populus deltoides* Genome 2002 45 1181 1188 10.1139/g02-094 12502265

[B16] Rooney AP Ward TJ Evolution of large ribosomal RNA multigene family in filamentous fungi: birth and death of a concerted evolution paradigm Proc Natl Acad Sci USA 2005 102 5084 5098 10.1073/pnas.0409689102 15784739 PMC555991

[B17] Amici A Rollo F The nucleotide sequence of the 5S ribosomal RNA gene of *Pyrenophora graminea* Nucl Acids Res 1991 19 5073 10.1093/nar/19.18.5073 1923773 PMC328812

[B18] Brown DD Carroll F Brown RD The isolation and characterization of a second oocyte 5S DNA from *Xenopus laevis* Cell 1977 12 1045 1056 10.1016/0092-8674(77)90168-4 563770

[B19] Bogenhagen DF Sakonju S Brown DD A control region in the center of the 5S RNA gene directs specific initiation of transcription II. The 3'border of the region Cell 1980 19 27 35 10.1016/0092-8674(80)90385-2 7357604

[B20] Bogenhagen DF Brown DD Nucleotide sequences in *Xenopus *5S DNA required for transcription termination Cell 1981 14 261 270 10.1016/0092-8674(81)90522-5 6263489

[B21] Komiya H Hasegawa M Takemura S Differentiation of oocyte- and somatic-type 5S rRNAs in animals J Biochem 1986 100 369 374 3782056 10.1093/oxfordjournals.jbchem.a121723

[B22] Pendás AM Moran P Freije JP Garcia-Vasquez E Chromosomal mapping and nucleotide sequence of two tandem repeats of Atlantic salmon 5S rDNA Cytogenet Cell Genet 1994 67 31 36 10.1159/000133792 8187548

[B23] Móran P Martínez JL Garcia-Vásquez E Pendás AM Sex linkage of 5S rDNA in rainbow trout (*Oncorhynchus mykiss*) Cytogenet Cell Genet 1996 75 145 150 10.1159/000134466 9040781

[B24] Sajdak SL Reed KM Phillips RB Intraindividual and interspecies variation in the 5S rDNA of coregonid fish J Mol Evol 1998 46 680 688 10.1007/PL00006348 9608050

[B25] Martins C Galetti PM Jr Organization of 5S rDNA in *Leporinus *fish species: two different genomic locations are characterized by distinct non-transcribed spacers (NTSs) Genome 2001 44 903 910 11681615 10.1139/g01-069

[B26] Wasko AP Martins C Wright JM Galetti PM Jr Molecular organization of 5S rDNA in fishes of the genus *Brycon* Genome 2001 44 893 902 11681614

[B27] Gornung E Colangelo P Annesi F 5S ribosomal RNA genes in six species of Mediterranean grey mullets: genomic organization and phylogenetic inference Genome 2007 50 787 795 10.1139/G07-058 17893718

[B28] Campo D Machado-Schiaffino G Horreo JL Garcia-Vazquez E Molecular organization and evolution of 5S rDNA in the genus *Merluccius *and their phylogenetic implications J Mol Evol 2009 68 3 208 216 10.1007/s00239-009-9207-8 19247563

[B29] Pasolini P Costagliola D Rocco L Tinti F Molecular organization of 5S rDNAs in Rajidae (Chondrichthyes): structural features and evolution of piscine 5S rRNA genes and nontranscribed intergenic spacers J Mol Evol 2006 62 564 574 10.1007/s00239-005-0118-z 16612546

[B30] Pinhal D Araki CS Gadig OBF Martins C Molecular organization of 5S rDNA in sharks of the genus *Rhizoprionodon*: insights into the evolutionary dynamics of 5S rDNA in vertebrate genomes Genet Res 2009 91 61 72 10.1017/S0016672308009993 19220932

[B31] Pinhal D Gadig OBF Martins C Genetic identification of the sharks *Rhizoprionodon porosus *and *R. lalandii *by PCR-RFLP and nucleotide sequence analyses of 5S rDNA Conserv Genet Res 2009 1 35 38 10.1007/s12686-009-9008-9

[B32] Nei M Hughes AL Tsuji K, Aizawa M, Sasazuki T Balanced polymorphism and evolution by the birth-and-death process in the MHC loci11th Histocompatibility workshop and conference 1992 Oxford: Oxford University Press 27 38

[B33] Nei M Gu X Sitnikova T Evolution by the birth-and-death process in multigene families of the vertebrate immune system Proc Natl Acad Sci USA 1997 94 7799 7806 10.1073/pnas.94.15.7799 9223266 PMC33709

[B34] Nei M Rogozin IB Piontkivska H Purifying selection and birth-and-death evolution in the ubiquitin gene family Proc Natl Acad Sci USA 2000 97 10866 10871 11005860 10.1073/pnas.97.20.10866 PMC27115

[B35] Vierna J Gonzalez-Tizon A Martinez-Lage A Long-term evolution of 5S ribosomal DNA seems to be driven by birth-and-death processes and selection in *Ensis *razor shells (mollusca: Bivalvia) Biochem Genet 2009 47 635 644 10.1007/s10528-009-9255-1 19633948

[B36] Pinhal D Gadig OBF Wasko AP Oliveira C Foresti F Martins C Discrimination of shark species by simple PCR of 5S rDNA repeats Genet Mol Biol 2008 31 361 365 10.1590/S1415-47572008000200033

[B37] Thorson TB Wooton RM Georgi TD Rectal gland of freshwater stingrays, *Potamotrygon *ssp. (Condrichthyes: Potamotrygonidae) Biol Bull 1978 154 508 516 10.2307/1541076 20693375

[B38] Compagno LJV Cook SF The exploitation and conservation of freshwater elasmobranchs: status of taxa and prospects for the future J Aquaric Aquat Sci 1995 7 62 90

[B39] Nishida K Phylogeny of the suborder Myliobatidoidei Memoirs of the Faculty of Fisheries, Hokkaido University 1990 37 1 108

[B40] Pieler T Hamm J Roeder RG The 5S gene internal control region is composed of three distinct sequence elements, organized as two functional domains with variable spacing Cell 1987 48 91 100 10.1016/0092-8674(87)90359-X 3791417

[B41] Posada D Crandall KA Modeltest: testing the model of DNA substitution Bioinformatics 1998 14 817 818 10.1093/bioinformatics/14.9.817 9918953

[B42] Martins C Wasko AP Oliveira C Porto-Foresti F Parise-Maltempi PP Wright JM Foresti F Dynamics of 5S rDNA in the tilapia (*Oreochromis niloticus*) genome: repeat units, inverted sequences, pseudogenes and chromosome loci Cytogenet Genome Res 2002 98 78 85 10.1159/000068542 12584446

[B43] Martins C Galetti PM Jr Two 5S rDNA arrays in neotropical fish species: is it a general rule for fishes? Genetica 2001 111 439 446 10.1023/A:1013799516717 11841188

[B44] Nederby-Nielsen J Hallenberg C Frederiksen S Sorensen PD Lomholt B Transcription of human 5S rRNA genes is influenced by an upstream DNA sequence Nucleic Acids Res 1993 26 3631 3636 10.1093/nar/21.16.3631 PMC309857 8367278

[B45] Suzuki H Sakurai S Matsuda Y Rat 5S rDNA spacer sequences and chromosomal assignment of the genes to the extreme terminal region of chromosome 19 Cytogenet Cell Genet 1996 72 1 4 10.1159/000134149 8565624

[B46] Lovejoy NR Stingrays, parasites, and historical biogeography: A closer look at Brooks et al's hypotheses for the origins of neotropical freshwater rays: Potamotrygonidae Syst Biol 1997 46 218 230 10.1093/sysbio/46.1.218

[B47] Lovejoy NR Bermingham E Martin AP South American rays came in with the sea Nature 1998 396 421 422 10.1038/24757

[B48] Underwood CJ Diversification of the Neoselachii (Chondrichthyes) during the Jurassic and Cretaceous Paleobiology 2006 32 2 215 235 10.1666/04069.1

[B49] Drouin G Expressed retrotransposed 5S rRNA genes in the mouse and rat genomes Genome 2000 43 213 215 10.1139/g99-100 10701135

[B50] Pelliccia F Barzotti R Bucciarelli E Rocchi A 5S rRNA and U1 snRNA genes: a new linkage type in the genome of a crustacean that has three different tandemly repeated units containing 5S rDNA sequences Genome 2001 44 331 335 11444690 10.1139/gen-44-3-331

[B51] Raskina O Belyayev A Nevo E Quantum speciation in *Aegilops*: molecular cytogenetic evidence from rDNA clusters variability in natural populations Proc Natl Acad Sci USA 2004 101 14818 14823 10.1073/pnas.0405817101 15466712 PMC522011

[B52] Raskina O Belyayev A Nevo E Activity of the *En/Spm *-like transposons in meiosis as a base for chromosome repatterning in a small, isolated, peripheral population of *Aegilops speltoides *Tausch Chromosome Res 2004 12 153 161 15053485 10.1023/b:chro.0000013168.61359.43

[B53] Martins C Ferreira IA Oliveira C Foresti F Galetti PM Jr A tandemly repetitive centromeric DNA sequence of the fish *Hoplias malabaricus *(Characiformes: Erythrinidae) is derived from 5S rDNA Genetica 2006 127 133 141 10.1007/s10709-005-2674-y 16850219

[B54] Kress H Bechler K Swida U Maletz S Evolution of 5S rRNA gene families in *Drosophila* Chromosome Res 2001 9 403 415 10.1023/A:1016787602583 11448042

[B55] Trontin JF Grandemange C Favre JM Two highly divergent 5S rDNA unit size classes occur in composite tandem array in European larch (*Larix deciduas *Mill.) and Japanese larch (*Larix kaempferi *(Lamb.) Carr.) Genome 1999 42 837 848 10584306

[B56] Falistocco E Passeri V Marconi G Investigations of 5S rDNA of *Vitis vinifera *L.: sequence analysis and physical mapping Genome 2007 50 927 938 10.1139/G07-070 18059555

[B57] Nei M Rooney AP Concerted and birth-and-death evolution in multigene families Annu Rev Genet 2005 39 121 152 10.1146/annurev.genet.39.073003.112240 16285855 PMC1464479

[B58] Pendás AM Móran P Martínez JL Garcia-Vásquez E Applications of 5S rDNA in Atlantic salmon, brown trout, and in Atlantic salmon x brown trout hybrid identification Mol Ecol 1995 4 275 276 10.1111/j.1365-294X.1995.tb00220.x 7735532

[B59] Aranishi F PCR-RFLP analysis of nuclear nontranscribed spacer for mackerel species identification J Agric Food Chem 2005 53 508 511 10.1021/jf0484881 15686394

[B60] Presa P Pardo BG Martínez P Bernatchez L Phylogeographic congruence between mtDNA and rDNA ITS markers in brown trout Mol Biol Evol 2002 19 2161 2175 12446808 10.1093/oxfordjournals.molbev.a004041

[B61] Ferreira IA Oliveira C Venere PC Galetti PM Jr Martins C 5S rDNA variation and its phylogenetic inference in the genus *Leporinus *(Characiformes: Anostomidae) Genetica 2006 129 253 257 16897448 10.1007/s10709-006-0005-6

[B62] Ohta T Dover GA The cohesive population genetics of molecular drive Genetics 1984 108 501 521 6500260 10.1093/genetics/108.2.501 PMC1202420

[B63] Liao D Gene conversion drives within genic sequences: converted evolution of ribosomal RNA genes in bacteria and archaea J Mol Evol 2000 51 305 17 11040282 10.1007/s002390010093

[B64] Anjard C Loomis WF Evolutionary analyses of ABC transporters of *Dictyostelium discoideum* Euk Cell 2002 1 643 52 10.1128/EC.1.4.643-652.2002 PMC117992 12456012

[B65] Eickbush TH Eickbush DG Finely orchestrated movements: evolution of the ribosomal RNA genes Genetics 2007 175 477 485 10.1534/genetics.107.071399 17322354 PMC1800602

[B66] Walsh JB Sequence-dependent gene conversion: can duplicated genes diverge fast enough to escape conversion? Genetics 1987 117 543 557 3692140 10.1093/genetics/117.3.543 PMC1203229

[B67] Insua A Freire R Ríos J Méndez J The 5S rDNA of mussels *Mytilus galloprovincialis *and *M. edulis*: sequence variation and chromosomal location Chromosome Res 2001 9 495 505 10.1023/A:1011636714052 11592484

[B68] Caradonna F Bellavia D Clemente AM Sisino G Barbieri R Chromosomal localization and molecular characterization of three different 5S ribosomal DNA clusters in the sea urchin *Paracentrotus lividus* Genome 2007 50 867 870 10.1139/G07-062 17893727

[B69] Fujiwara M Inafuku J Takeda A Watanabe A Fujiwara A Kohno S Kubota S Molecular organization of 5S rDNA in bitterlings (Cyprinidae) Genetica 2009 135 355 365 10.1007/s10709-008-9294-2 18648989

[B70] Eirín-López JM Gonzalez-Tizon AM Martinez A Mendez J Birth-and-death evolution with strong purifying selection in the histone H1 multigene family and the origin of orphon H1 genes Mol Biol Evol 2004 21 1992 2003 10.1093/molbev/msh213 15254261

[B71] Eirín-López JM González-Romero R Dryhurst D Méndez J Ausió J Pontarotti P Long-term evolution of histone families: old notions and new insights into their diversification mechanisms across eukaryotes Evolutionary Biology: Concept, Modeling, and Application 2009 Berlin, Heidelberg: Springer-Verlag 139 162

[B72] Piontkivska H Rooney AP Nei M Purifying selection and birth-and-death evolution in the histone H4 gene family Mol Biol Evol 2002 19 689 97 11961102 10.1093/oxfordjournals.molbev.a004127

[B73] Nikolaidis N Nei M Concerted and nonconcerted evolution of the Hsp70 gene superfamily in two sibling species of nematodes Molec Biol Evol 2004 21 498 505 14694072 10.1093/molbev/msh041

[B74] Zhang Z Inomata N Yamazaki T Kishino H Evolutionary history and mode of the amylase multigene family in *Drosophila* J Mol Evol 2003 57 702 709 10.1007/s00239-003-2521-7 14745539

[B75] Hartmann S Nason JD Bhattacharya D Extensive ribosomal DNA genic variation in the columnar cactus *Lophocereus* J Mol Evol 2001 53 124 134 11479683 10.1007/s002390010200

[B76] Mayol M Rossello JA Why nuclear ribosomal DNA spacers (ITS) tell different stories in *Quercus* Mol Phyl Evol 2001 19 167 176 10.1006/mpev.2001.0934 11341800

[B77] Márquez LM Miller DJ MacKenzie JB van Oppen MJH Pseudogenes contribute to the extreme diversity of nuclear ribosomal DNA in the hard coral Acropora Mol Biol Evol 2003 20 1077 1086 10.1093/molbev/msg122 12777522

[B78] Rooney AP Mechanisms underlying the evolution and maintenance of functionally heterogeneous 18S rRNA genes in apicomplexans Mol Biol Evol 2004 21 1704 1711 10.1093/molbev/msh178 15175411

[B79] Kellogg EA Appels R Intraspecific and interspecific variation in 5S RNA genes are decoupled in diploid wheat relatives Genetics 1995 140 325 343 7635297 10.1093/genetics/140.1.325 PMC1206559

[B80] McLysaght A Hokamp K Wolfe KH Extensive genomic duplication during early chordate evolution Nat Genet 2002 31 200 204 10.1038/ng884 12032567

[B81] Dehal P Boore JL Two rounds of whole genome duplication in the ancestral vertebrate PLoS Biol 2005 3 e314 10.1371/journal.pbio.0030314 16128622 PMC1197285

[B82] Meyer A Van de Peer Y From 2R to 3R: evidence for a fish-specific genome duplication (FSGD) BioEssays 2005 27 937 945 10.1002/bies.20293 16108068

[B83] Martins C Wasko AP Williams CR Organization and evolution of 5S ribosomal DNA in the fish genome Focus on Genome Research 2004 Hauppauge: Nova Science Publishers 335 363

[B84] Sambrook J Russel DW Molecular Cloning: A Laboratory Manual 2001 New York: Cold Spring Harbor Laboratory Press

[B85] Wegnez M Denis H Mazabraud A Clerot JC RNA accumulation during oogenesis of the dogfish *Scyliorhinus caniculus*. Biochemical research on oogenesis Dev Biol 1978 62 99 111 10.1016/0012-1606(78)90095-7 620878

[B86] Rocco L Costagliola D Fiorillo M Tinti F Stingo V Molecular and chromosomal analysis of ribosomal cistrons in two cartilaginous fish, *Taeniura lymma *and *Raja montagui *(Chondrichthyes, Batoidea) Genetica 2005 123 245 253 10.1007/s10709-004-2451-3 15954495

[B87] Edgar RC MUSCLE: multiple sequence alignment with high accuracy and high throughput Nucl Acids Res 2004 32 5 1792 1797 10.1093/nar/gkh340 15034147 PMC390337

[B88] Hall TA BioEdit: a user-friendly biological sequence alignment editor and analysis program for Windows 95/98/NT Nucl Acids Symp Ser 1999 41 95 98

[B89] Guindon S Gascuel O A simple, fast and accurate algorithm to estimate large phylogenies by maximum likelihood Syst Biol 2003 52 696 704 10.1080/10635150390235520 14530136

[B90] Guindon S Lethiec F Duroux P Gascuel O PHYML Online - a web server for fast maximum likelihood-based phylogenetic inference Nucl Acids Res 2005 33 557 559 10.1093/nar/gki352 PMC1160113 15980534

[B91] Swofford DL PAUP* Phylogenetic analysis using parsimony (*and other methods). Version 4b10 2002 Sunderland: Sinauer Associates

[B92] Felsenstein J Confidence limits on phylogenies: an approach using the bootstrap Evolution 1985 39 783 791 10.2307/2408678 28561359

[B93] Huelsenbeck JP Ronquist F Nielsen R Bollback JP Bayesian inference of phylogeny and its impact of evolutionary biology Science 2001 294 2310 2314 10.1126/science.1065889 11743192

[B94] Ronquist F Huelsenbeck JP MrBayes 3: Bayesian phylogenetic inference under mixed models Bioinformatics 2003 19 1572 1574 10.1093/bioinformatics/btg180 12912839

[B95] Tamura K Dudley J Nei M Kumar S MEGA4: Molecular Evolutionary Genetics Analysis (MEGA) software version 4.0 Mol Biol Evol 2007 24 1596 1599 10.1093/molbev/msm092 17488738

[B96] Librado P Rosas J DnaSP v5: A software for comprehensive analysis Bioinformatics 2009 25 1451 1452 10.1093/bioinformatics/btp187 19346325

