# Supplementary material for: The 5S rDNA family evolves through concerted and birth-and-death evolution in fish genomes: an example from freshwater stingrays
Source: BMC Evol Biol. 2011 May 31;11:151. doi: 10.1186/1471-2148-11-151 (PMC3123226; doi:10.1186/1471-2148-11-151)
Supplement: Additional file 1 — Final alignment of nucleotide sequences encompassing the class I and class II 5S rRNA genes from the three Potamotrygonidae stingrays. Species are referred to as follows: Pfalkneri = Potamotrygon falkneri, Pmotoro = P. motoro, Paireba = Paratrygon aiereba. Dots represent sequence identity, gray shadowed nucleotides are indicative of distinctive sites between 5S genes found in the two 5S rDNA arrays. The internal control regions (A box, IE and C box) are highlighted in black. a, 5S rRNA genes class II; b, 5S rRNA genes class I. [file 1471-2148-11-151-S1.PDF]

Additional File 1. Final alignment of nucleotide sequences encompassing the class I and class II 5S rRNA genes from the three Potamotrygonidae stingrays. Species are referred to as follows: Pfalkneri = *Potamotrygon falkneri*, Pmotoro = *P. motoro*, Paireba = *Paratrygon aiereba*. Dots represent sequence identity, gray shadowed nucleotides are indicative of distinctive sites between 5S genes found in the two 5S rDNA arrays. The internal control regions (A box, IE and C box) are highlighted in black. a, 5S rRNA genes class II; b, 5S rRNA genes class I.

```
             +44                                                                         +120
Pfalkneri01  CTCGGAAGCT AAGCAGGCTC AGGCCTGGTT AGTACTTGGA TGGGAGACCG GCTGGGAATA CCAGGTGCCG TAGGCTT
Pfalkneri02  .......... .......... .......... .......... .......... C........G .......... .......
Pfalkneri03  .......... .......... .......... .......... .......... C......... .......... .......
Pmotoro01    .......... .......... .......... .......... .......... C......... .......... .......
Pmotoro02    .......... .......... .......... .......... .......... C......... .......... .......
Pmotoro03    .......... .......... .......... .......... .......... C......... .......... .......
Pmotoro04    .......... .......... .......... .......... .......... C......... .......... .......
Pmotoro05    .......... .......... .......... .......... .......... C......... .......... .......   a
Pmotoro06    .......... .......... .......... .......... .......... C......... .......... .......
Paiereba06   .......... .......... ....G..... .......... .......... C......... .......... .......
Paiereba01   .......... .......... .......... .......... C......... C......... .......... .......
Paiereba02   .......... .......... .......... .......... .......... C..A...... .......... .......
Paiereba03   .......... .......... .......... .......... .......... C......... .......... .......
Paiereba04   ......T... .......... .......... .......... .A........ C......... .......... .....C.
Paiereba05   ......T... .......... .......... G......... .......... C......... .......... .......
Paiereba07   .......... .......... .......... .......... .......... C......... .......... .......
Pmotoro11    ..T....... ......A... .......... .......... ........T. C......... ........A. .......
Pmotoro12    ..T....... ......A... .......... .......... ........T. C......... ........A. .......
Pmotoro13    ..T....... ......A... .......... .......... ........T. C......... ........A. .......
Pmotoro14    ..T....... ......A... .......... .......... ........T. C......... ........A. .......
Pmotoro15    ..T....... ..A...A... .......... .......... ........T. C......... ........A. .......
Pmotoro16    ..T....... ......A... .......... .......... ........T. C......... ........A. .......
Pfalkneri21  ..T....... ..A...A... .......... .......... ........T. C......... ........A. .......   b
Pfalkneri22  ..T....... ......A... .......... .......... ........T. C......... ........A. .......
Pfalkneri23  ..T....... ......A... .......... .......... ........T. C.C....... ........A. .......
Pfalkneri24  ..T....... ......A... .......... .......... .....N..T. C.C....... ........A. .......
Pfalkneri25  ..T....... ......A... .......... .......... ........T. C......... ........A. .......
Pfalkneri26  ..T....... ......A... .......... .......... ........T. C......... ........A. .......
Paiereba31   .......... ......A... .......... .......... .....A.... C......... ........A. .......
Paiereba32   .......... ......A... .......... .......... .....A.... C......... ........A. .......
```
