# Supplementary material for: The 5S rDNA family evolves through concerted and birth-and-death evolution in fish genomes: an example from freshwater stingrays
Source: BMC Evol Biol. 2011 May 31;11:151. doi: 10.1186/1471-2148-11-151 (PMC3123226; doi:10.1186/1471-2148-11-151)
Supplement: Additional file 2 — NTS class I and NTS class II nucleotide sequences from the stingray species included in this study. a) Nucleotide sequence alignment of short NTS repetitions of class I; (b) partial nucleotide sequence alignments of long NTS repetitions of class II obtained from the stingrays genome. Species are referred to as follows: Pfalkneri = Potamotrygon falkneri, Pmotoro = P. motoro, Paireba = Paratrygon aiereba. Dots represent conserved nucleotides and hyphens report indels. The microrepetition TCCC expanded in the P. aiereba genome is indicated in gray shading. Dots represent conserved bases and hyphens report indels. [file 1471-2148-11-151-S2.PDF]

Additional file 2. NTS class I and NTS class II nucleotide sequences from the stingray species included in this study. (a) Nucleotide sequence alignment of short NTS repetitions of class I; (b) partial nucleotide sequence alignments of long NTS repetitions of class II obtained from the stingrays genome. Species are referred to as follows: Pfalkneri = *Potamotrygon falkneri*, Pmotoro = *P. motoro*, Paireba = *Paratrygon aiereba*. Dots represent conserved nucleotides and hyphens report indels. The microrepetition TCCC expanded in the *P. aiereba* genome is indicated in gray shading. Dots represent conserved bases and hyphens report indels.

a)

```
Pmotoro11    TTACTGCCAG CAGGGGCTGC CCACCAGGCA GGCTGCGGGC AGTGCCGGCC CCGAGGCAGC CAACCTTTTG GCCGGAGGGA
Pmotoro12    .......... .......... .......... .......... .......... .......... .......... ..........
Pmotoro13    .......... .......... .......... .......... .......... .......... .......... ..........
Pmotoro14    .......... .......... .......... .......... .......... .......... .......... ..........
Pmotoro15    .......... .......... .......... .......... .......... .......... .......... ..........
Pmotoro16    .......... .......... .......... .......... .......... .......... .......... ..........
Pfalkneri21 .......... .......... .......... .......... .......... .......... .......... ..........
Pfalkneri22 .......... .......... .......... .......... .......... .......... .......... ..........
Pfalkneri23 .......... .......... .......... .......... .......... .......... .......... ..........
Pfalkneri24 .......... .......... .......... .......... .......... .......... .......... ..........
Pfalkneri25 .......... .......... .......... .......... .......... .......... .......... ..........
Pfalkneri26 .......... .......... .......... .......... .......... .......... .......... ..........
Paiereba31   ..G....... .......... .......... ......A... .......... .......... .......... ..........
Paiereba32   ..G....... .......... .......... ......A... .......... .......... .......... ..........

Pmotoro11    GGCTGAGGGA CTTTGCTGTG TGCTCTCTGG GAGATGTCGT TGTGGCAAGA TCGACCCGGA AGCGCCAAGC GCACCATCTG
Pmotoro12    .......... .......... .......... .......... .......... .......... .......... ..........
Pmotoro13    .......... .......... .......... .......... .......... .......... .......... ..........
Pmotoro14    .......... .......... .......... .......... .......... .......... .......... ..........
Pmotoro15    .......... .......... .......... .......... .......... .......... .......... ..........
Pmotoro16    .......... .......... .......... .......... .......... .......... .......... ..........
Pfalkneri21 .......... .......... .......... .......... .......... .......... .......... ..........
Pfalkneri22 .......... .......... .......... .......... .......... .......... .......... ..........
Pfalkneri23 .......... .......... .......... ...G...... .......... .......... .......... ..........
Pfalkneri24 .......... .......... .......... ...G...... .......... .......... .......... ..........
Pfalkneri25 .......... .......... .......... .......... .......... .......... .......... ..........
Pfalkneri26 .......... .......... .......... .......... .......... .......... .......... ..........
Paiereba31   .......... .......C.. .......... .......... .......... .......... .......... ..........
Paiereba32   .......... .......C.. .......... .......... .......... .......... .......... ..........

Pmotoro11    TGCTGCCAGC AATATCCACC AACAAGTGCC CAGCGCAGGA TCTCTGGTGT GTCAATGGGG TTGACCCCAA ACACTACGTC
Pmotoro12    .......... .......... .......... .......... .......... .......... .......... ..........
Pmotoro13    .......... .......... .......... .......... .......... .......... .......... ..........
Pmotoro14    .......... .......... .......... .......... .......... .......... .......... ..........
Pmotoro15    .......... .......... .......... .......... .......... .......... .......... ..........
Pmotoro16    .......... .......... .......... .......... .......... .......... .......... ..........
Pfalkneri21 .......... .......... .......... .......... .......... .......... .......... ..........
Pfalkneri22 .......... .......... .......... .......... .......... .......... .......... ..........
Pfalkneri23 .......... .......... .......... .......... .......... .......... .......... ..........
Pfalkneri24 .......... .......... .......... .......... .......... .......... .......... ..........
Pfalkneri25 .......... .......... .......... .......... .......... .......... .......... ..........
Pfalkneri26 .......... .......... .......... .......... .......... .......... .......... ..........
Paiereba31   .......... .......... .......... .......... ......C..A C......... .......... ........C.
Paiereba32   .......... .......... .......... .......... ......C..A C......... .......... ........C.

Pmotoro11    AGTGTTTCCT TCC------- -CTCCCTCCG CCTCTGACCA AAGCGGAAAG CCAGGTCCCT CTGACCCGTC AGTCTGAATT
Pmotoro12    .......... ...------- -......... .......... .......... ........-. .......... ..........
Pmotoro13    .......... ...------- -......... .......... .......... .......... .......... ..........
Pmotoro14    .......... ...------- -......... .......... .......... .......... .......... ..........
Pmotoro15    .......... ...------- -......... .......... .......... .......... .......... ..........
Pmotoro16    .......... ...------- -......... .......... .......... .......... .......... ..........
Pfalkneri21 .......... ...------- -......... .......... .......... .......... .........T ..........
Pfalkneri22 .......... ...------- -......... .......... .......... .......... .........T ..........
Pfalkneri23 .......... ...------- -......... .......... .......... .......... .......... ..........
Pfalkneri24 .......... ...------- -......... .......... .......... .......... .......... ..........
Pfalkneri25 .......... ...------- -......... .......... .......... .......... .......... ..........
Pfalkneri26 ........T. C..------- --........ .......... .......... .......... .......... ..........
Paiereba31   .......... ...TTCCCTC C......... .......... .......... .......... .......... ..........
Paiereba32   .......... ...TTCCCTC C......... .......... .......... ........TC T......... ..........

Pmotoro11    GGCCAGAAAT GTGTGCAGAG ATTGCCTATG GCCATACTAG CCTGAATCG 359
Pmotoro12    .......... .A........ .......... .......... ......... 358
```

```
Pmotoro13   .......... .......... .......... .......... ......... 361
Pmotoro14   .......... .A........ .......... .......... ......... 360
Pmotoro15   .......... .A........ .......... .......... ...-.....A 359
Pmotoro16   .......... .A........ .......... .......... ...-.....A 344
Pfalkneri21 ......G... .A........ .......... .......... .........A 360
Pfalkneri22 ......G... .A........ .......... .......... .........A 361
Pfalkneri23 .......... .......... .......... .......... ......... 356
Pfalkneri24 .......... .A........ .......... .......... ......... 360
Pfalkneri25 .......... .......... .......... .......... ......... 361
Pfalkneri26 .......... .A........ .......... .......... ......... 358
Paiereba31  ........G. ...G..GC.. .G........ .......... ......... 369
Paiereba32  ........G. .A.G..GC.. .G........ .........A ......... 366
```

b)

```
Pfalkneri01 TTGCTGTT-T TGCTGCTGCT GCTGTTGCTG CTGCTGCCG- --------CA CTGAACAGAT GTCCGCAGTA GGAGCTGCTC
Pfalkneri02 .......A-. .......... ....C..... .........- --------.. .......... .......... ..........
Pfalkneri03 ........-. .......... ....C..... .........- --------.. .......... .......... ..........
Pmotoro01   ........-. .......... ..-------- ---------- ---------. .......... .......... ..........
Pmotoro02   ........-. .......... ..-------- ---------- ---------. .......... .......... ..........
Pmotoro03   ........-. .......... ..-------- ---------- ---------. .......... .......... ..........
Pmotoro04   ........-. .........A ....C..T.. ......---- --------.. .......... .......... ..........
Pmotoro05   ........-. .......... ..-------- ---------- ---------. .......... .......... ..........
Pmotoro06   ........-. .......... ..-------- ---------- ---------. .......... .......... ..........
Paiereba01  ......C.GC .......... ---------- ---------- -------C.T .......... .......... ..........
Paiereba02  ......G.GC .......... ....C..... ....------ -------C.T .......... .....G.... .......A..
Paiereba03  ......C.GC .......... ..-------- ---------- -------C.T .......... .......... ..........
Paiereba04  ......C.GC .......... ....C..... .......T.C TGCTGCTC.T .......... .......... ..........
Paiereba05  ......C.-C .......... ..-------- ---------- -GCTGCTC.T .......... .......... ..........

Pfalkneri01 TCTTCTTTTC AGTCCGACTC TGACATACAC TGTCGGCACT GCGCTGCTCA ATCAGCCCAG AAACGCAGGC ACCCGGCTCC
Pfalkneri02 .......... .......... .......... .......... .....T.... C....T.... .......A.. ..........
Pfalkneri03 .......... .......... .......... .......... .T...T.... C......... .......A.. ..........
Pmotoro01   ....T..A.. ......C... G......... .C........ .T...T.... C......... .......A.. ..........
Pmotoro02   ....T..... ......C... G......... .C........ .....T.... C......... .......A.. ..........
Pmotoro03   ....T..... ......C... G......... .C........ .....T.... C......... .......A.. ..........
Pmotoro04   ....T..... ......C... G......... .C........ .....T.... C......... .......A.. ..........
Pmotoro05   ....T..... ......C... G......... .C........ .....T.... C......... .......A.. ..........
Pmotoro06   ....T..... ......C... G......... .C........ .....T.... C......... .......A.. ..........
Paiereba01  .......... ......C... .......G.. .C........ .....T.... C......... .......A.. ....T.....
Paiereba02  .......A.. ......C... .......... .C........ .A...T.... C......... .......A.. ....T.....
Paiereba03  .......... ......C... .......... .C........ .....T.... C....A.... .......A.. .?..T.....
Paiereba04  .......... ......C... .......G.. .C........ .....T.... C....A.... .......A.. ....T.....
Paiereba05  .......... ......C... .......... .C....A... .....T.... C......... .......A.. ....T.....

Pfalkneri01 TGCTGTCAAG GCACACGAAT ACAAAAATGT GCTTTTGCGA AGCAGTCAGC TGCACCTGCA GCATTATCCG TTGTAATTGC
Pfalkneri02 .......... .......... .......... .......A.. .......... .......... ........A. .C........
Pfalkneri03 .......... .......... .......... .......... .......... .......... ........A. .C........
Pmotoro01   .......... .......... ....G..... .......... G......... .....T.... ...C....-. .CC.C.....
Pmotoro02   .......... .....G.... ....G..... ....?..... G...C..... .....T.... ...C..G.-. .CC.C.....
Pmotoro03   .......... .....G.... ....G..... .......... G....C.... .....T.... ...C..G.-. .CC.C.....
Pmotoro04   ......T... .....G.... ....G..... .......... G....C.... .....T.... ...C..G.-. .CC.C.....
Pmotoro05   .......... .....G.... ....G..... .......... G....C.... .....T.... ...C..G.-. .CC.C.....
Pmotoro06   .......... .....G.... ....G..... .......... G....C.... .....T.... ...C..G.-. .CC.C.....
Paiereba01  .......... .....G.... ....G..... .........T G.-------- -...T.C--- -----.GT-. C..C..G...
Paiereba02  .......... .....G.... .T..G..... .........T G.-------- -...T.C--- -----.GT-. C..C..G...
Paiereba03  .......... .....G.... ....G..... .........T G.-------- -...T.C--- -----.AT-. C..C..G...
Paiereba04  .......... .....G.... ....G..A.. .........T G...C..... .....T.--- -----.CT-. C..C..G...
Paiereba05  .......... .....G.... ....T..... .........T G.-------- -...T.C--- -----.GT-. C..C..G...

Pfalkneri01 TTCCTCCTCC TCGCACCACT TCAGACGCAC GCCCGCACTC GCAACTTGCC ACACGCTCTT CTTTACCCTC TTTTCTCCAC
Pfalkneri02 .......... ......T... .......... .......... ..T....... .......... .......... ..........
Pfalkneri03 .......... ......T... .......... .......... .......... .......... .......... ..........
Pmotoro01   ..T....... .......... .......... .......... .......... G......... .......... ..........
Pmotoro02   .......... .......... .....G.... .......... .......... G......... .......... ..........
Pmotoro03   .......... .......... .......... .......... .......... G......... .......... ..........
Pmotoro04   .......... .......... .......... .......... ....T..... G......... .......... .........G
Pmotoro05   .......... .......... .......... .......... .......... G......... .......... ..........
Pmotoro06   .......... .......... .......... .......... .......... G......... .......... ..........
Paiereba01  .......... .......... .......... .......... .......... ...A...... ....C..... ..........
Paiereba02  .......... .......... .......... .......... .......... ...A...... ....C..... ..........
Paiereba03  .......... .......... .......... .......... .......... ...A...... ....C..... ..........
Paiereba04  .......... .......... .......... .......... .......... ...A...... ....C..... ..........
Paiereba05  .......... .......... .......... .......... .......... ...A...... ....C..... ..........

Pfalkneri01 TCCTCTGCCT TCGCCTCCAA CTTTGCGCTT CCAGCCCCTT ---------- --------CA CTCCCCTCTC ACCTGC----
Pfalkneri02 .......... .......... .......... .......... ---------- --------.. ..G....... ......----
Pfalkneri03 .......... .......... .......... .......... ---------- --------.. .......... ......----
Pmotoro01   .......... .......... .......... .......... CACTCACACC CTACCCTT.. ....A..... ......----
Pmotoro02   .......... .......... .......... .......... CACTCACACC CTACCCTT.. ....A..... ......----
Pmotoro03   .......... .......... .......... .......... CACTCACACC CTACCCTT.. ....A..... ......----
Pmotoro04   .......... ...A...... .......... .......... TAGTCACACC CTACCCTT.. ......C... ......----
Pmotoro05   .......... .......... .......... .......... CACTCACACC CTACCCTT.. .......... ......----
Pmotoro06   .......... .......... .......... .......... CACTCACACC CTACCCTT.. .......... ......----
```

```
Paiereba01  .......... .......... .......... .......... CACTCACATC CTACCCTA.. .C.TT..... C...CTAACT
Paiereba02  .......... .......... T......... .......... CACTCACATT CTACCCTA.. .C.TT..... C...CTAACT
Paiereba03  .......... .......... .......... .......... CACTCACATC CTACCCTA.. .C.TT..... C...CTAACT
Paiereba04  .......... .......... .......... .......... CACTCACATC CTACCCTA.. .C.TT..... C...CTAACT
Paiereba05  .......... .......... .......... .......... CACTCACATC CTACCCTA.. .C.TT..... C...CTAACT

Pfalkneri01 ---CCTCCTC ACCATGCGCT GCTTCACCGC ACTACACTGC TTGCCGCTCA CCATAGCCTA CACCTCACAA TCACCGCTCT
Pfalkneri02 ---....... .......... .......... .......... .......... .......... .......... ..........
Pfalkneri03 ---....... .......... .......... .........T .......... .......... .......... ......A...
Pmotoro01   ---....... .......... .......T.. .......... .......... .........C .......... ..........
Pmotoro02   ---....... .......... .......T.. .......... .......... ......G..C ..A....... ..........
Pmotoro03   ---....... .......... .......T.. .......... .......... .........C .......... ..........
Pmotoro04   ---....... .......... .G.......A .......... .......... .........C .......... ..........
Pmotoro05   ---....... .......... .......... .......... .......... .........C .......... ..........
Pmotoro06   ---....... .......... .......... .......... .......... .........C .......... ..........
Paiereba01  TGC......A .......... .......... .......... .......... ....T...AC .......... ..........
Paiereba02  TGC....... .......... .......... .......... .......... ....T...AC ..G....... ..........
Paiereba03  TGC....... .......... ......TG.. .......... .......... ....T.T.AC ..G....... ..........
Paiereba04  TGC....... .......... .......... .......... .......... ....T...AC .......... ..........
Paiereba05  TGC....... .......... .......... .......... .......... ....T.T.AC ..G....... ......A...

Pfalkneri01 TGCCTCACAC GCACACTCAC TCACTCACAC ACACACACAC ACACACACAA GCGCGCGCGC GCACACAAAA CGCGCTCACT
Pfalkneri02 .......... .......... .......... .......... .......... .......... .......... .A........
Pfalkneri03 .......... .......... .......... .......... .......... .......... .......... ..........
Pmotoro01   .......... .......... .......... .......... ...AG.G--C .......... .....-.... .A........
Pmotoro02   .......... .......... .......... .......... ...AG.G--C .......... .....-.... .A........
Pmotoro03   .......... .......... .......... .......... ...AG.G--C .......... .....-.... .A........
Pmotoro04   .......... .....A.... .......... .......... ...AG.G--C .......... ...A.-.... .A........
Pmotoro05   .......... .......... .......... .........A G.--G.G--C .......... A....-.... .A........
Pmotoro06   .......... .......... .......... .........A G.--G.G--C .......... A....-.... .A........
Paiereba01  .T.......- ---------. ........T. ..TT...... ....G...GC A...A..GA. .....-.... .A...A....
Paiereba02  ?T.......- ---------. .......... ..TT...... ....G...GC A...----A. .....-.... .A...A....
Paiereba03  .T.......- ---------. .......... ..TT...... ....G...GC A...----A. .....-.... .A...A....
Paiereba04  .T.......- ---------. ........T. ..TT...... ....G...GC A...A...A. .....-.... .A...A....
Paiereba05  .T.......- ---------. ........T. ..TT...... ....G...GC A...A...A. .....-.... .A...A....

Pfalkneri01 CACCTCTAAA CTGCCCTGAC AGCCGCTCGA CTCTCTTTCC CTT-GATTCG CCTTTACACA CCCTT-CTGG
Pfalkneri02 .......... .......... .......... ........-. ...-...... G......... .....-....
Pfalkneri03 .......... .........G .......... .......... ...-...... .......... .....-...T
Pmotoro01   .......... G......... ..G....... .......G.. ...-...... .......... .....-...C
Pmotoro02   .......... .......... .AG....... .......G.. ...-...... .......... .....-...C
Pmotoro03   ..A....... .......... .......... .......G.. ...-...... .......... .....-...C
Pmotoro04   .......... A....TG..G ..A....... ......GG.. ...-...... .......... ..T.?-.G.C
Pmotoro05   .......... ...G...... ..G....... .......G.. ...G...... G......... .....T...C
Pmotoro06   .......... ...G...... ..G....... .......G.. ...G...... .......... .....-....
Paiereba01  .......C.- .......... G......... .......... ...-C..... T........C A....AT..C
Paiereba02  .......C.- .......... G....A.... .......... ...-C..... .........C A....AT..C
Paiereba03  .......C.- .......... G......... .......... ...-C..... .........C A....AT..C
Paiereba04  .......C.- ...G...... G......... .......... ...-C..... .........C A....AT..C
Paiereba05  ..A....C.- ...G...... G......... .......... ...-C..... G........C A....AT..C
```
