# Supplementary material for: The 5S rDNA family evolves through concerted and birth-and-death evolution in fish genomes: an example from freshwater stingrays
Source: BMC Evol Biol. 2011 May 31;11:151. doi: 10.1186/1471-2148-11-151 (PMC3123226; doi:10.1186/1471-2148-11-151)
Supplement: Additional file 3 — Alignment of 5S rRNA gene sequences from several vertebrates. The 5S gene of lampreys and diverse bony fish orders are majority-rule consensus (MRC) sequences obtained from data source listed in Additional file 1. (s) somatic type; (o) oocyte type. Sequences generated in the present study are underlined. [file 1471-2148-11-151-S3.PDF]

Additional File 3. Alignment of 5S rRNA gene sequences from several vertebrates. The 5S gene of lampreys and diverse bony fish orders are majority-rule consensus (MRC) sequences obtained from data source listed in the Supplementary file 1. (s) somatic type; (o) oocyte type. Sequences generated in the present study are underlined.

```
Petrom MRC CTCGGAAGCT AAGCAGGGTC GAGCCTGGTT AGTACTTGGA TGGGAGACCG CCTGGGAATA CCAGGTGTTG TAGGCTT-
Pfalkneri01.......... .......C.. AG........ .......... .......... G......... .......CC. .......-
Pfalkneri02.......... .......C.. AG........ .......... .......... .........G .......CC. .......-
Pfalkneri03.......... .......C.. AG........ .......... .......... .......... .......CC. .......-
Pmotoro01  .......... .......C.. AG........ .......... .......... .......... .......CC. .......-
Pmotoro02  .......... .......C.. AG........ .......... .......... .......... .......CC. .......-
Pmotoro03  .......... .......C.. AG........ .......... .......... .......... .......CC. .......-
Pmotoro04  .......... .......C.. AG........ .......... .......... .......... .......CC. .......-
Pmotoro05  .......... .......C.. AG........ .......... .......... .......... .......CC. .......-
Pmotoro06  .......... .......C.. AG........ .......... .......... .......... .......CC. .......-
Paiereba06 .......... .......C.. AG..G..... .......... .......... .......... .......CC. .......-
Paiereba01 .......... .......C.. AG........ .......... C......... .......... .......CC. .......-
Paiereba02 .......... .......C.. AG........ .......... .......... ...A...... .......CC. .......-
Paiereba03 .......... .......C.. AG........ .......... .......... .......... .......CC. .......-
Paiereba04 .......T.. .......C.. AG........ .......... .A........ .......... .......CC. .....C.-
Paiereba05 .......T.. .......C.. AG........ G......... .......... .......... .......CC. .......-
Paiereba07 .......... .......C.. AG........ .......... .......... .......... .......CC. .......-
Pmotoro11  ..T....... ......AC.. AG........ .......... ........T. .......... .......CA. .......-
Pmotoro12  ..T....... ......AC.. AG........ .......... ........T. .......... .......CA. .......-
Pmotoro13  ..T....... ......AC.. AGN....... .......... ........T. .......... .......CA. .......-
Pmotoro14  ..T....... ......AC.. AG........ .......... ........T. .......... .......CA. .......-
Pmotoro15  ..T....... ..A...AC.. AG........ .......... ........T. .......... .......CA. .......-
Pmotoro16  ..T....... ......AC.. AG........ .......... ........T. .......... .......CA. .......-
Pfalkneri21..T....... ..A...AC.. AG........ .......... ........T. .......... .......CA. .......-
Pfalkneri22..T....... ......AC.. AG........ .......... ........T. .......... .......CA. .......-
Pfalkneri23..T....... ......AC.. AG........ .......... ........T. ..C....... .......CA. .......-
Pfalkneri24..T....... ......AC.. AG........ .......... .....N..T. ..C....... .......CA. .......-
Pfalkneri25..T....... ......AC.. AG........ .......... ........T. .......... .......CA. .......-
Pfalkneri26..T....... ......AC.. AG........ .......... ........T. .......... .......CA. .......-
Paiereba31 .......... ......AC.. AG........ .......... .....A.... .......... .......CA. .......-
Paiereba32 .......... ......AC.. AG........ .......... .....A.... .......... .......CA. .......-
RlalII     .......... .......... .G........ .......... .......... .......... .......C.. ..A....-
RporII     .........C .......... .G........ .......... .......... .......... ..C....C.. ..A....-
RlalI      .......... ......AC.. AG........ .......... .......... .......... .......CA. .......-
RporI      .......... ......AC.. AG........ .......... .......... .......... .......CA. .......-
Asuper     .......-.. ..A...AC.. AG-....... .......... ........T. .......... .......CA. .......-
Gcuvier    .......... ......AC.. AG........ .......... .......... .......... .......CC. .......-
Scanic     .......... ......AT.. AG........ .......... .......... .......... .......CA. .......-
Doxyrin    .......... .......C.. AG........ .......... .......... .......... .......CC. .......-
Rmir       .......... .......C.. AG........ .......... .......... .......... .......CC. .......-
Rpoly      .......... .......C.. AG........ .......... .......... .......... .......CC. .......-
Rclav      .......... .......C.. AG........ .......... .......... .......... .......CC. .......-
RasteII    .......... .......C.. AG........ .......... .......... .......... .......CC. .......-
RasteI     ......TC.C .......TAA .G.......C .......... .......... .....A.... T......C.. ..A....-
Tlymma     .......... .......C.. AG........ .......... .......... .......... .......CC. .......-
AcipMRC    ...A...... .....AC..T .G..T..... .......... .........A .......... T..A...C.. .....A.T
CypriMRC   .......... .......T.T .G........ .......... .......... .......... .......C.. ..A....-
CyprinoMRC .......... .......... .G........ .......... ........T. .......... .......C.. ..A....-
GadiMRC    .......... .......... .G........ .......... .......... .......... .......C.. ..A....-
GastMRC    ..T....... .......... .T........ .....C.... .....C..T. .......... .......C.. .......-
PerciMRC   .......... .......... .G.......C .......... .......... .......... ......CC.. ..A....-
PleuroMRC  .......... .......... .G........ .......... .......... .......... .......C.. ..A....-
SalmoMRC   .......... .......... .G........ .......... ........T. .......... .......C.. ..A....-
Silu       .........A ......A..T .G........ .........T ........T. AA........ ........C. .......-
AnguiMRC   .......... .......... .G........ .......... .......... .......... .......CA. ..A....-
Tetraodo   .......... .......... .G.....C.. .......... .......... .......... .......C.. ..A....-
BcephI     ..T....... .......... .G........ .......... ........T. .......... .......CC. .GA....-
BSPII      ..T....... .........T .G....CA.. ...T...T.. A.......T. .......... .......C.. ..A....-
Lobt       .......... ........C. .G........ .......... .......... .......... .......C.. ..A....-
Calleg     ...A...... .......C.T .G........ .......... ........T. .......... .......C.. .......-
Adavi      ...A...... ...G.....T .G.TT.A... .......... ........T. .......... .......C.. .......-
Ajapo      .......... .........T .G........ .......... ........T. ..C......- ..C....C.. .......T
Xtrop(s)   .......... ........A. .G........ .......... .......... .......... ..T....C.. .......-
Xtrop(o)   .......... ........A. .G..T..... .....C.... .......... .......... .......... .......-
Grioba     .......... .......... .G........ .......... .......... .......... .......C.. .......-
Nvirid     ...A....T. .......... .G........ .....C.... .......... ...T...... .......C.. .......-
Iiguana    .......... .......... .G........ .......... .......... .......... ..G....C.. .......-
Ggallus    .......... .......... .G........ .......... .........T .......... ..G....C.. .......-
Mfasc      .......... .......... .G........ .......... .......... .......... ..G....C.. .......-
Rnorv      .......... .......... .G........ .......... .......... .......... ..G....C.. .......-
Mmusc      .......... .......... .G........ .......... .......... .......... ..G....C.. .......-
Hsapiens   .......... .......... .G........ .......... .......... .......... ..G....C.. .......-
Sramster   .......... .......... .G........ .......... .......... .......... ..G....C.. .......-
Btaurus    .......... .......... .G........ .......... .......... .......... ..G....C.. .......-
```
