# Supplementary material for: The 5S rDNA family evolves through concerted and birth-and-death evolution in fish genomes: an example from freshwater stingrays
Source: BMC Evol Biol. 2011 May 31;11:151. doi: 10.1186/1471-2148-11-151 (PMC3123226; doi:10.1186/1471-2148-11-151)
Supplement: Additional file 4 — Compilation of information regarding 5S rDNA nucleotide sequence in vertebrates. 5S rDNA information of several vertebrate groups (except stingrays sequences) were retrieved from GenBank/EMBL/DDBJ and used in the present study. [file 1471-2148-11-151-S4.PDF]

Additional file 4. Compilation of information regarding 5S rDNA nucleotide sequence in vertebrates. 5S rDNA information of several vertebrate groups (except stingrays sequences) were retrieved from GenBank/EMBL/DDBJ and used in the present study.

Major groups and taxa	Species	GenBank Accession Number	References
**Fishes**			
Petromyzontiformes	*Lethenteron japonicum*	D00076, X04308, X04309	Komiya et al (1986)
	Lethenteron reissneri	X13038	Qi et al (1988)
Carcharhiniformes	*Rhizoprionodon lalandii class II*	FJ517177-FJ517195	Pinhal et al. (2009a)
	Rhizoprionodon porosus class II	FJ517196-FJ517211	Pinhal et al. (2009a)
	Rhizoprionodon lalandii class I	FJ517166-FJ517172	Pinhal et al. (2009a)
	Rhizoprionodon porosus class I	FJ517173-FJ517176	Pinhal et al. (2009a)
	Scyliorhinus caniculus	M24954	Wegnez et al. (1978)
	Galeocerdo cuvier	FJ539130	Pinhal et al. (2009a)
Lamniformes	*Alopias superciliosus*	FJ539131	Pinhal et al. (2009a)
Rajiformes	*Dipturus oxyrinchus*	DQ020573	Pasolini et al. (2006)
	Raja miraletus	DQ020567	Pasolini et al. (2006)
	Raja polystigma	DQ020563	Pasolini et al. (2006)
	Raja clavata	DQ020559	Pasolini et al. (2006)
	Raja asterias type II	DQ020553	Pasolini et al. (2006)
	Raja asterias type I	DQ020547	Pasolini et al. (2006)
	Taeniura lymma	AY278251	Pasolini et al. (2006)
	Raja montagui(polystigma)	AY278250	Pasolini et al. (2006)
Acipenseriformes	*Acipenser baerii*	AJ744968-AJ744977	Robles et al. (2005)
	Acipenser brevirostrum	AJ745060-AJ745070	Robles et al. (2005)
	Acipenser fulvescens	AJ745057-AJ745059	Robles et al. (2005)
	Acipenser gueldenstaedtii	AJ745033-AJ745037	Robles et al. (2005)
	Acipenser naccarii	AJ744955-AJ744967	Robles et al. (2005)
	Acipenser oxyrinchus	AJ744991-AJ744996	Robles et al. (2005)
	Acipenser ruthenus	AJ745038-AJ745047	Robles et al. (2005)
	Acipenser sinensis	AJ745017-AJ745028	Robles et al. (2005)
	Acipenser stellatus	AJ745048-AJ745056	Robles et al. (2005)
	Acipenser transmontanus	AJ745003-AJ745016	Robles et al. (2005)
Cypriniformes	*Acheilognatus melanogaster*	AB072205,AB072206	Fujiwara et al. (2009)
	Acheilognathus rhombeus	AJ745003-AJ745016, AB072197, AB072198	Inafuku et al. (2000); Fujiwara et al. (2009)
	Acheilognatus tabira	AB015591, AB072193, AB072194, AB072201,AB072202	Inafuku et al. (2000); Fujiwara et al. (2009)
	Acheilognatus typus	AB072204	Fujiwara et al. (2009)
	Rhodeus ocellatus	AB072199, AB072203	Fujiwara et al. (2009)
	Rhodeus sericeus	AB072195, AB072196	Fujiwara et al. (2009)
	Tanakia tanago	AB072200	Fujiwara et al. (2009)
	Cyprinus carpio	AB015590	Inafuku et al. (2000)

	Carassius auratus langsdorfi	AB001492-AB001503, AB007776-AB007784	Marukami and Fujitani (1998)
	Misgurnus fossilis	V00647-V00648, X56631-X56642	Mashkova et al. (1981); Sedman et al. (1989)
Cyprinodontiformes	*Lebias fasciata*	AY302496-AY302498	Tigano et al. (2004)
Gadiformes	*Arctogadus glacialis*	AY955254	Unpublished
	Gadus morhua	AY150824	direct submission
	Merlangius merlangus	AY150825	direct submission
	Merluccius gayi	AF335600, FJ196631	direct submission and Campo et al. (2009)
	Merluccius merluccius	AF335598, FJ196634	direct submission and Campo et al. (2009)
	Merluccius paradoxus	AF335597, AF335597, FJ196635, FJ196636	direct submission and Campo et al. (2009)
	Merluccius polli	AF335599, FJ196637, FJ196638	direct submission and Campo et al. (2009)
	Merluccius senegalensis	FJ196640	Campo et al. (2009)
	Merluccius capensis	FJ196630	Campo et al. (2009)
	Merluccius angustimanus	FJ196625	Campo et al. (2009)
	Merluccius australis	FJ196626	Campo et al. (2009)
	Merluccius productus	FJ196639	Campo et al. (2009)
	Merluccius albidus	FJ196623, FJ196624	Campo et al. (2009)
	Merluccius hubbsi	FJ196632, FJ196633	Campo et al. (2009)
	Merluccius bilinearis	FJ196627-FJ196629	Campo et al. (2009)
	Micromesistius poutassou	AY150820	direct submission
	Molva dipterygia	AY150828	direct submission
	Molva molva	AY150827	direct submission
	Pollachius pollachius	AY150826	direct submission
	Synbranchus marmoratus	AY271269	Messias et al. (2003)
	Trisopterus esmarkii	AY150822	direct submission
	Trisopterus luscus	AY150823	direct submission
	Trisopterus minutus	AY150821	direct submission
Gasterosteiformes	*Gasterosteus aculeatus*	n.p.	Rocco et al. 1999
Perciformes	*Danio rerio*	AF213516, AF213517	Gornung et al. (2000)
	Liza ramada	AY695111	Rossi et al. (2005)
	Micropterus salmonoides	L49397	Deiana et al.(2000)
	Oreochromis niloticus	AF176349, AF176497-AF176505, AF478461-AF478462, AY763289-AY763351	Martins et al. (2000); Martins et al. (2002)
	Sparus aurata	AY330701-AY330702	Sola et al. (2003)
Pleuronectiformes	*Lepidorhombus whiffianogis*	AF335601	direct submission
	Paralichthys olivaceus	AB154836-AB154839	direct submission
	Reinhardtius hippoglossoides	n.p.	Céspedes et al. (1999)
	Solea solea	n.p.	Céspedes et al. (1999)
Salmoniformes	*Salmo gairdnerii*	J01861	Komiya & Takemura (1979)
	Salmo salar	S73106, S73107	Pendas et al. (1994)
	Coregonus artedi	U84590-U84605	Sajdak et al. (1998)
	Coregonus zenithicus	U84606-U84614	Sajdak et al. (1998)
Siluriformes	*Ictalurus punctatus*	L49398	Unpublished
Anguilliformes	*Anguilla Anguilla*	DQ339039-DQ339041	Pichiri et al. (2006)

	Anguilla rostrata	DQ323734	Pichiri et al. (2006)
Tetraodontiformes	*Tetraodon nigroviridis*	AJ245808	Roest Crollius et al. (2000)
Characiformes	*Brycon cephalus type I*	AF250529	Wasko et al. (2001)
	Brycon SP. type II	AF250536	Wasko et al. (2001)
	Leporinus obtusidens	AF284747	Martins and Galleti (2001b)
	Leporinus cf. elongatus	AF284737-AF284742	Martins and Galleti (2001b)
	Leporinus elongatus	AF284728-AF284731	Martins and Galleti (2001b)
	Leporinus friderici	AF284743-AF284746, AF284748	Martins and Galleti (2001b)
	Leporinus obtusidens	AF284732-AF284736, AF284747	Martins and Galleti (2001b)
	Hoplias malabaricus	AY624052-AY624061	Martins et al. (2006)
**Amphibians**	*Cryptobranchus alleganiensis*	AB066113	Fujiwara et al. (2006)
	Andrias davidianus	AB066110	Fujiwara et al. (2006)
	Andrias japonicus	AB066108	Fujiwara et al. (2006)
	Xenopus tropicalis(s)	X12622	Nietfeld et al. (1998)
	Xenopus tropicalis(o)	X12624	Nietfeld et al. (1998)
	Gastrotheca riobambae	M74438	Del Pino et al. (1992)
	Notophtalmus viridescens	M13611	Kay and Gall (1981)
**Reptiles**	*Iguana iguana*	M10817	Roy and Enns (1976)
**Avian**	*Gallus gallus*	X01309	Pace et al. (1974)
**Mammals**	*Macaca fascicularis*	AF193591	Jensen and Frederiksen (2000)
	Rattus norvegicus	X83746	Frederiksen et al. (1997)
	Mus musculus	X71804	Hallenberg et al. (1994)
	Homo sapiens	V00589	Forget and Weissman (1967)
	Syrian hamster	J00063	Hart and Folk (1982)
	Bos taurus	X57170	Szymanski et al. (1992)
